# Evaluation of a New Lightweight EEG Technology for Translational Applications of Passive Brain-Computer Interfaces

**DOI:** 10.3389/fnhum.2022.901387

**Published:** 2022-07-14

**Authors:** Nicolina Sciaraffa, Gianluca Di Flumeri, Daniele Germano, Andrea Giorgi, Antonio Di Florio, Gianluca Borghini, Alessia Vozzi, Vincenzo Ronca, Fabio Babiloni, Pietro Aricò

**Affiliations:** ^1^BrainSigns Srl, Rome, Italy; ^2^Department of Molecular Medicine, Sapienza University of Rome, Rome, Italy; ^3^College of Computer Science and Technology, Hangzhou Dianzi University, Hangzhou, China; ^4^Department of Computer, Control, and Management Engineering “Antonio Ruberti”, Sapienza University of Rome, Rome, Italy

**Keywords:** passive-BCI, EEG, water-based electrodes, workload, vigilance, stress, human factors

## Abstract

Technologies like passive brain-computer interfaces (BCI) can enhance human-machine interaction. Anyhow, there are still shortcomings in terms of easiness of use, reliability, and generalizability that prevent passive-BCI from entering real-life situations. The current work aimed to technologically and methodologically design a new gel-free passive-BCI system for out-of-the-lab employment. The choice of the water-based electrodes and the design of a new lightweight headset met the need for easy-to-wear, comfortable, and highly acceptable technology. The proposed system showed high reliability in both laboratory and realistic settings, performing not significantly different from the gold standard based on gel electrodes. In both cases, the proposed system allowed effective discrimination (AUC > 0.9) between low and high levels of workload, vigilance, and stress even for high temporal resolution (<10 s). Finally, the generalizability of the proposed system has been tested through a cross-task calibration. The system calibrated with the data recorded during the laboratory tasks was able to discriminate the targeted human factors during the realistic task reaching AUC values higher than 0.8 at 40 s of temporal resolution in case of vigilance and workload, and 20 s of temporal resolution for the stress monitoring. These results pave the way for ecologic use of the system, where calibration data of the realistic task are difficult to obtain.

## Introduction

The human–machine interaction involves all the processes of interaction and communication between a human user and a machine. It aims to make the human even more “understandable to the machine” by systems that can detect and classify human feelings, emotions, and cognitive states (Di Nardo et al., 2020). In recent times, more and more attention arose around the use of brain signals to detect the human state, that is the passive brain-computer interfaces (BCI). BCI was born for clinical needs and progressively moved from translating intentional (actions) to unintentional (mental states) brain outputs, thus, from active to passive-BCI (Zander et al., 2009). The passive-BCI does not aim to voluntarily control the external world but to allow the surroundings to automatically adapt their behavior to the actual subject's mental states (Zander and Kothe, 2011; Aricò et al., 2018). The impact of this new aspect of the BCI is clear for safety-critical environments, where the mental state of users is usually correlated to the probability of making errors or, more generally, to human factors (Aricò et al., 2017b; Zander et al., 2017). The monitoring of the user-state is just one of the countless applications of passive-BCI that have been resumed in 5 categories. The additions to user-state monitoring are safety and security, training, education, cognitive improvement, gaming, and entertainment (Erp et al., 2012).

Despite the growing interest in passive-BCI applications, there is still a gap between theory and practice. On the one hand, the advantage of using a human-centered design is clear, since it could facilitate the operator's work and reduce safety risks (Ke et al., 2021). On the other hand, ethical and technical limitations prevent the growth of passive-BCI technology within operational contexts: ease of use, reliability, and generalizability are the key characteristics affecting the end-users perception and choice of a passive-BCI system (Douibi et al., 2021).

### Technical Limitations of Passive-BCI Systems

In this context, electroencephalography (EEG) is the most used method to measure the electrical activity of the brain. It is usually preferred, thanks to its portability, non-invasiveness, and high temporal resolution (Roman-Gonzalez, 2012). However, a traditional passive-BCI setup requires the use of Ag/AgCl electrodes that are in contact with the scalp through the electrolytic gel. This strongly limits the ease of use (Webster, 2009). In fact, in place of excellent signal quality, these recording systems required the scrubbing of the skin to lower the electrode impedance before using electrolytic gel. This is a time-consuming activity that takes 20 min or more depending on the number of electrodes used. Extra time is also needed because the subject should wash the hair to remove gel residues. This approach and gel-based electrodes are still considered the gold standard. However, the gap between the new water and gel electrodes is narrowing (Volosyak et al., 2010; Chi et al., 2012; Lopez-Gordo et al., 2014; Di Flumeri et al., 2019a). Water-based electrodes consisted of felt or similar tissue soaked in water or saline solution to connect the electrode to the skin (Müller-Putz and Wriessnegger, 2021). This makes both setup and clean-up faster and easier, as well as more comfortable without affecting the quality of the signal (Volosyak et al., 2010). In this regard, different portable and gel-free EEG recording systems have been employed in several studies: the tested electrodes were able to guarantee the same quality levels as the gel electrodes, along with significantly faster setup and higher comfort (Volosyak et al., 2010; Amaral et al., 2017; Vourvopoulos et al., 2017; Di Flumeri et al., 2019a).

Despite this, the wearable EEG devices available on the market are a step behind other wearable devices. A recent analysis of the current market found 28 companies developing wired and wireless EEG equipment (Jamil et al., 2021). However, it has not yet been possible to converge comfort with pleasant appearance, effective recording, and adaptability to different operating contexts.

Despite the wide range of recording systems, the time-to-market estimate made 10 years ago for passive-BCI systems has not yet been met (Erp et al., 2012). This is also because the reliability of a passive-BCI system depends not only on hardware but also on software aspects. On the one hand, the recording technology should guarantee ease of use without adversely affecting signal quality. On the other hand, the passive-BCI system should be accurate and not bore the user with frequent calibrations. This aspect depends on the suitability of the algorithm implemented in the system. First, signal processing techniques should be adapted for out-of-the-lab use. For example, the low number of channels expected for wearable EEG and the need for high reactivity disfavor the most widely used signal correction methods, such as Independent Component Analysis (Makeig et al., 1995). Furthermore, the more the number of pre-processing steps, the higher the computational effort required, resulting in larger computational modules and faster battery consumption. In addition, for online use, all methods that need to know the entire data (e.g., normalization methods) cannot be used. Once clean EEG signals have been obtained, further steps have to be performed to obtain features that can identify the user's mental state. Spectral features can be efficiently calculated and can provide real-time feedback. Such features are usually used to train machine learning or deep learning algorithms (Aricò et al., 2016). These methods must be reliable, but must not require frequent calibration, ideally zeroing it out with an unsupervised approach (Schultze-Kraft et al., 2016). Although unsupervised approaches may allow greater generalizability of the system, supervised methods are preferred as they allow significantly higher accuracies to be achieved (Blankertz et al., 2016). Better generalization results can be achieved using a cross-task approach. An open issue related to the use of passive-BCI during a realistic task is the lack of specific calibration tasks able to elicit a specific mental state in the subject. Well-designed laboratory tasks can be effective for calibration use in a cross-task approach.

To summarize, EEG technology and calibration of the passive-BCI system remain the two main gaps for out-of-the-lab use.

### Targeted Human Factors

Drivers, air traffic controllers, surgeons, athletes, and pilots represent some of the users who could benefit most from the use of passive-BCI systems during both training and operational conditions (Aricò et al., 2017a; Zander et al., 2017; Alimardani and Hiraki, 2020). In general, the common denominator of these application areas is human performance: passive-BCI could allow obtaining information on the user's psychophysiological state, in terms of different human factors, to develop solutions for improving performance and safety. Building on this, three human factors have been targeted in the current work: mental workload, stress, and vigilance.

The mental workload has been chosen due to its relevant role in safety-critical applications (Young et al., 2015). In general, the mental workload quantifies the mental resources used during a task and is correlated with the performance (Wickens, 2008). Extremely high (i.e., overload), while extremely low (i.e., underload) workloads have been associated with poorer performances. Therefore, a passive BCI system could intervene to detect a fatal overload/underload condition. Assessment of mental workload by EEG provides a reliable and objective measure that can be used for this purpose in different laboratory and realistic contexts (Zhou et al., 2021).

Stress is another relevant human factor in many operational domains (Aricò et al., 2017b). One of the definitions of stress is “a state that occurs when demand outstrips coping strategies” (Hobfoll and Shirom, 1993). High task demand, frustration, time pressure, uncontrollability, and negative judgment are typical stressors that adversely affect performance by altering the cognitive processes underlying decision-making, attention, and memory (Skoluda et al., 2015). Stress assessment by EEG takes advantage of the higher temporal resolution and the possibility of direct access to the activity of the central nervous system, measuring stress levels continuously and without interfering with human activities (Borghini et al., 2020). Moreover, there are several examples of real-time measures of stress based on EEG signals using machine learning methods (Saeed et al., 2015; Attallah, 2020; Halim and Rehan, 2020; Hekmatmanesh et al., 2021; Katmah et al., 2021).

Finally, monitoring levels of vigilance has proven to be a key aspect in several fundamental environments (Neigel et al., 2020). The concept of vigilance belongs to the broader domain of attention. A physiological decrease in vigilance over time is associated with performance degradation, such as slower reaction times and loss of situation awareness, whereas optimal performance is ensured by an adequate level of activation throughout the task (Parasuraman et al., 1998). EEG measures have been used as features for machine learning models to monitor vigilance levels in different contexts (Sebastiani et al., 2020; Kamrud et al., 2021; Li and Chung, 2022).

In this framework, the present work aims to evaluate a new lightweight EEG technology for translational use of passive-BCI. In particular, the system based on water-based electrodes was tested in terms of impedances, artifacts, usability, and human factors assessment and was compared with a gel-based equivalent that was used as a gold standard. To analyze the reliability and generalizability of the proposed system, it has been tested during two studies focusing on laboratory and realistic settings, respectively.

## Materials and Methods

### Experimental Participants

Twenty healthy subjects (29.3 ± 5.12 years old) took part in the study: 8 women and 12 men were recruited voluntarily. Informed consent was obtained from each subject and all the data were pseudorandomized to prevent any association with the subject's identity. The experiments were conducted following the principles outlined in the 1975 Declaration of Helsinki, as revised in 2000. Experiments were approved by the Ethical Committee of the Sapienza University of Rome. Due to the involvement in a realistic driving experiment, a driver's license and the ability to drive a car with a manual transmission were required as inclusion criteria.

### Experimental Set-Up

**Figure 1:**
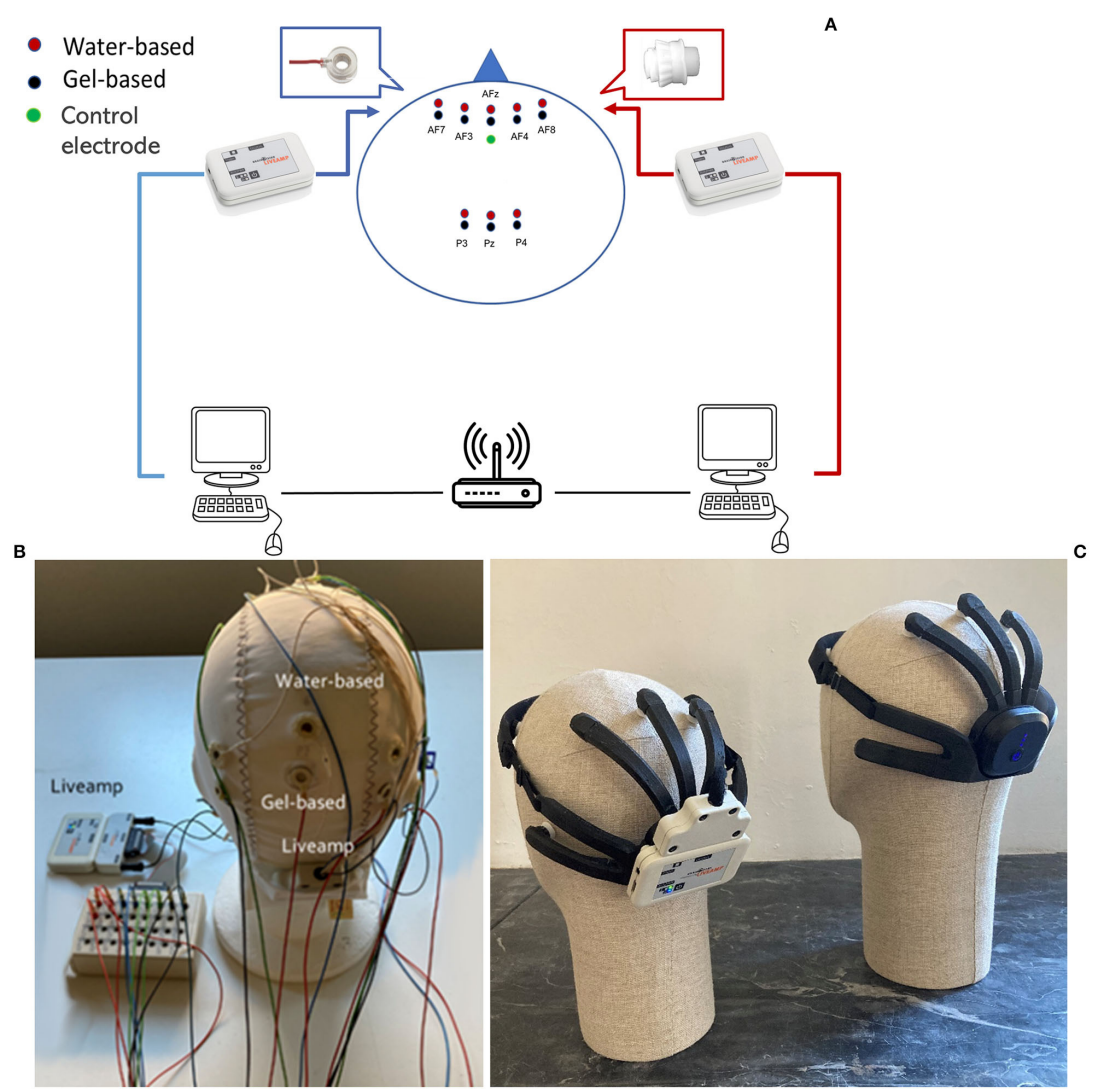
Scheme of the experimental set-up. **(A)** A scheme of the position of the electrodes and instrumental set-up. Water-based electrodes are shown in red, gel-based electrodes in blue, and the control electrode in green. **(B)** The actual experimental set-up was used for the EEG sensors comparison. **(C)** Mindtooth Touch EEG headset, designed for working with water-based electrodes (right), and modified version equipped with the LiveAmp amplifier (left).

Gel and water-based electrodes were used for this experiment (Figure 1A). The gel-based electrodes were traditional Ag/AgCl electrodes produced by EasyCap GmbH (Woerthsee-Etterschlag, Germany). The water-based electrodes consisted of open-celled, hydrophilic, and highly absorbent cylindrical sponges produced by Brain Products GmbH (Gilching, Germany). It hardens when dry and becomes soft and expandable when wet. The porous material is intended to soak aqueous electrolyte solutions (1–2% sodium chloride solutions are used as electrolytes). The electrolyte solution is used to accomplish an easy electrically conducting connection to the skin of the subject. While this mechanism of aqueous electrolyte is similar to the one of gel electrodes, the big advantage of aqueous electrolyte is that it does not leave significant amounts of residuals in the hair; the water in the electrolyte evaporates over time just leaving tiny amounts of salt on the skin that is removed with the next hair wash. To slow down the evaporation of water, a soft pedestal hermetically sealed the chamber. The sponges are integrated into a holder that can be easily connected to the electrode base using a bayonet twist lock (i.e., sponge holder). When the sponge holder is screwed into its counterpart on the electrode, the other end of the sponge comes into contact with an electrode pellet. The electrode pellet is made of a conductive plastic that is coated with a thin layer of silver/silver chloride (Ag/AgCl), which is an electrically stable and widely used electrode material.

Both gel-based and water-based electrodes have been positioned at a 1.5 cm of distance between each other on a standard EEG cap (Figure 1B). This has been done to maximize the hypothesis to ideally record from the same point and to guarantee a minimum distance to not generate short circuits between near electrodes The investigated scalp positions corresponded to AFz, AF3, AF4, AF7, AF8, Pz, P3, and P4 of the 10–10 International System. These specific channels have been chosen for both theoretical and practical reasons. On the one hand, these positions should guarantee the assessment of the identified human factors covering the required brain areas as known from previous evidence (please refer to “Targeted Human Factors” in the Section Introduction). On the other hand, a low number of electrodes is necessary to foster ease of use. It was also inserted a gel-based “Control” electrode, at a 1.5 cm of distance to the AFz position to quantify the possible difference between near couples of electrodes because of the distance among them.

For each set of electrodes, we used a LiveAmp amplifier (Brain Products GmbH, Gilching, Germany) (Figure 1A). Each amplifier has been connected to a homemade recording software developed in Python and based on Lab Streaming Layer (LSL) communication protocol. This software was run on two tablets with Microsoft Windows 10 operating system. The two tablets were connected to the same intranet, by using a wired LAN connection. Thanks to the LSL protocol and the LAN wired connection, it was possible to guarantee a perfect synchronization among all the connected devices, unless there is a lag of two times the inverse of the sampling rate (i.e., 8 ms).

In addition, the water-based electrodes have also been tested when embedded in the Mindtooth Touch EEG headset (referred to as “New Structure” in the Section Results) to maximize the comfort for the user, sensor contact, and easiness to fit (Figure 1C). In particular, the front part of the headset hosts the five anterior-frontal EEG sensors and is designed to fit the user's forehead. To maximize comfort, it is elastic and made of a combination of flexible materials. The front part has removable padding made in polyurethane (PU) foam to further improve comfort. The rear part hosts the ground and reference sensors, the three parietal EEG sensors, and the EEG amplifier. Ground and reference sensors are fitted to two separate “fingers” on the mastoids. The “fingers” of the three parietal EEG sensors are made as a separate part that is fitted and fixed to the rear part of the headset. All the parietal EEG sensors' holders have been prolonged and fitted to a rotating end-piece “a fingertip” to make it easier to adapt to all head shapes, split the hair, and touch the scalp. The hardware within the EEG amplifier has been developed by Brain Products (GmbH, Gilching, Germany). For the specific purposes of this experiment, the original amplifier of the Mindtooth Touch headset has been replaced with a LiveAmp amplifier, so as to not induce any bias due to different hardware.

### EEG Acquisition and Preprocessing

Once arrived, each subject was asked to wear the EEG standard cap with both the electrode types. Sponges of water-based electrodes were first disinfected using a solution of water (70%) and isopropanol (30%) and then partially soaked with 1% of NaCl saline solution. The quantity of saline solution was limited to the needed quantity to have a good signal quality, but not too much to induce dripping, and so as to generate possible shortcuts with near gel-based electrodes. At this point, the conductive gel was applied to all the gel-based electrodes in a limited quantity, so as to not generate any shortcuts between near electrodes. Reference electrodes of both the systems were put on the earlobe, and the ground on the left mastoid, by assuring even there a minimum distance to not have any shortcuts. Before starting the recording, the impedances have been brought below 40 kΩ (Ferree et al., 2001) and 100 kΩ (Kappenman and Luck, 2010) threshold for gel and water-based sensors, respectively.

The EEG signal was first band-pass filtered with a fifth-order Butterworth filter in the interval 2–30 Hz. The blink artifacts were detected using the Reblinca method (Di Flumeri et al., 2016) and were corrected by leveraging the ocular component estimated through a multi-channel Wiener Filter (MWF) (Somers et al., 2018). EEG signals were segmented into epochs of 1 s, and the threshold criterion (±80 μV) was applied for artifact rejection (Hubbard et al., 2019). This conservative value has been preferred to 100 μV, the default value suggested within the EEGlab toolbox, because just one criterium has been adopted (Delorme and Makeig, 2004).

### Experimental Protocol

The experimental protocol consisted of a sequence of laboratory (Study 1) and realistic (Study 2) tasks. They have been proceeded by two rest conditions: the subjects were asked to stay 1 min with closed eyes and 1 min with open eyes looking at a white cross on a screen. The closed eyes condition has been used to compute the individual alpha frequency (IAF) value (Klimesch, 1999). Since the alpha peak is mainly prominent during rest conditions, the subjects were asked to keep their eyes closed for a minute before starting the experiment. Such a condition was then used to estimate the IAF value specifically for each subject. The open eyes condition has been used to compare the two systems in terms of spectral features (Lopez-Gordo et al., 2014).

Therefore, the subjects were asked to familiarize themselves with the tasks. During task familiarization, they wore the Mindtooth Touch headset embedded with water-based electrodes. In this time interval of about 40 min, the impedances have been evaluated and saved at the end of each sample task. These values have been compared with the impedances of water-based electrodes recorded while placed on the standard EEG cap to compare the stability of the signals against the Mindtooth Touch Headset. Moreover, at the end of each experimental condition during both studies 1 and 2, impedance values of gel and water-based sensors were evaluated and saved to compare the stability of the impedance values between the two kinds of sensors.

#### Study 1: Test Laboratory Tasks

The aim of Study 1 was to analyze the performance of the light EEG system during standard controlled laboratory tasks like Multitasking and psychomotor vigilance task (PVT) as well as to provide the data for calibrating the passive-BCI system for a cross-task application.

**Figure 2:**
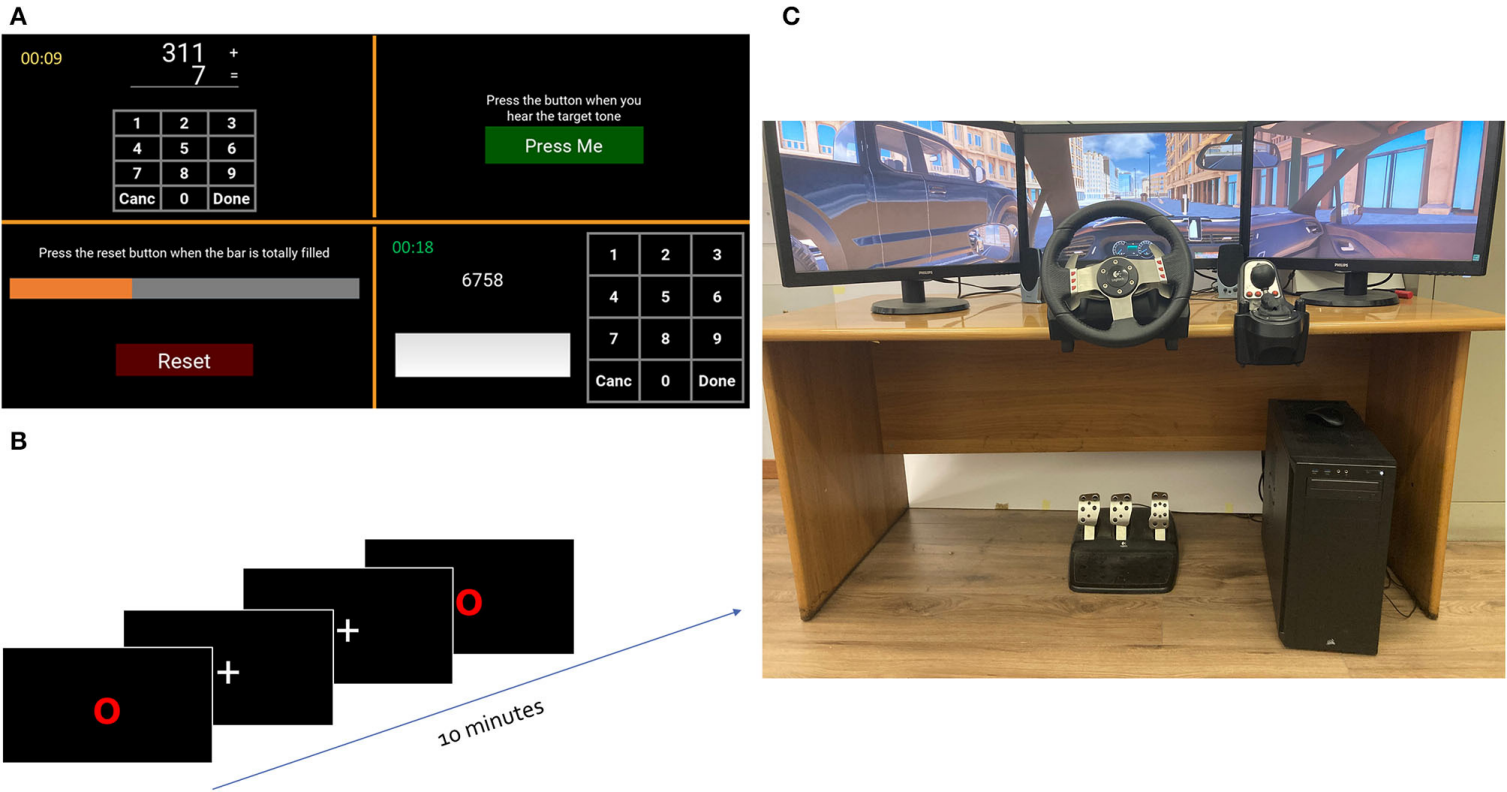
Experimental settings. **(A)** Multitasking application screen. In clockwise order, they are the auditory monitoring, the phone number entry task, the visual monitoring, and the mental arithmetic task. **(B)** Psychomotor vigilance task (PVT). During a 10-min stimulation, the subjects are required to press the space bar on the keyboard only when the red circle appears. **(C)** Car simulator. Car Driving simulator where the experimental participant is sitting.

First, the subjects were asked to accomplish the multitasking. The multitasking application comprises a set of four concurrent cognitive tasks of varying difficulty presented *via* split-screen (Figure 2A). The four chosen tasks are:

1. Mental arithmetic (left-up): The addition results must be entered into the numeric keypad. As the difficulty increases, the number of digits (from 1 to 3) and carryover digits (from 0 to 2) increases.
2. Auditory monitoring (right-up): A target tone must be identified between two tones of different frequencies emitted at regular intervals. As the difficulty increases, the target tone and distractor tone increase in similarity.
3. Visual monitoring (left-down): A horizontal fill bar should be reset as soon as it becomes full. As the difficulty increases, the fill rate increases.
4. Phone number entry task (right-down): A number must be entered on a keypad. As the difficulty increases, the number of digits to be entered increases (from 4 to 10).

According to the literature, performing several concurrent tasks compared to the single-task approach induces an increase in mental workload (Comstock, 1992; Wetherell and Sidgreaves, 2005). In addition, increased difficulty induces a greater perception of time pressure and frustration, resulting in increased stress. Therefore, the subjects were asked to perform the four tasks individually. In this case, a 30-s condition was performed for the auditory monitoring, visual monitoring, phone number entry task, easy mental arithmetic task, and hard mental arithmetic task. After that, subjects performed the four concurrent tasks (multitasking phase) at the same time. In this regard, 7 levels of 1 min multitasking at increasing difficulty have been performed. To confirm workload and stress manipulation, performance measures have been recorded. We hypothesized that the subject experienced the following: (i) low mental workload during the single task execution; (ii) high mental workload during the multitasking execution; and (iii) increasing stress during the 7 levels of multitasking at increasing difficulty.

At the end of multitasking, the subjects were asked to perform the PVT to elicit different levels of vigilance. The PVT is a computerized version of the Wilkinson and Houghton task (Wilkinson and Houghton, 1982) aiming to analyze the decrease of vigilance in 10 min (Figure 2B). During this task, subjects are asked to press the space bar on the keyboard as quickly as possible in response to a red circle appearing on the screen for 1 s after a fixation cross. The inter-stimulus intervals range randomly from 2 to 5 s. According to the literature (Molina et al., 2019), this monotonous task can induce a decrement of vigilance in participants, therefore we hypothesized that the experimental sample experienced: (i) a high level of vigilance during the first 2 mins (levels PVT1 and PVT2); (ii) low level of vigilance during the last 2 mins (levels PVT9 and PVT10).

#### Study 2: Test Realistic Driving Task

The aim of Study 2 was to analyze the performance of the light EEG system during realistic driving and to test the passive-BCI system calibrated with data recorded during the laboratory tasks. The car simulator is a typical real car setup (seat and dashboard with the steering wheel, gearshift, and all the common commands), while the virtual environment is reproduced through three 27″ screens (Figure 2C). This configuration was enough to make the driving environment immersive for the participants, thanks to the high realism of the driving environment. The implemented exercise allowed the elicitation of variations in workload, stress, and vigilance. The task for the participants was to follow the navigator instructions shown on the central screen.

In particular, for the mental workload assessment, two repetitions of easy and hard exercises have been generated. In the first repetition, the exercise was set in a circuit, and in the second repetition, it was set in an urban context with traffic. The level of difficulty was changed by acting on the number of curves, intersections, bottlenecks, pedestrians crossing the street, and the number of cars. Easy and hard conditions have been randomized among subjects.

For the stress scenarios, participants were instructed to perform the same two-run repetitions just completed, but with three more stressors:

1. Time pressure: Participants have a limited time to make the same route. A chronometer was used to show the remaining time.
2. White coat effect: An operator was just behind the driver, taking notes of errors committed.
3. External noise: Heavy urban traffic noise has been presented during the exercise.

Finally, the subjects were asked to complete the scenario used to induce decrement in vigilance. It was a 10-mins drive on the highway, without any traffic, in which the participant had to drive all the time in first gear in complete silence (i.e., the sound of the car has been muted).

The car simulator was able to generate a log file with all the specific events that could happen within each scenario execution. Among the possible available events, collisions of the car with anything in the simulated world have been considered performance indicators. In particular, the more the number of collisions, the more difficult or stressful the specific level was. We analyzed the number of collisions for the easy, hard, and stressful conditions.

### Performed Analysis

Taking into account the spatial and temporal variability of the EEG signal, standard procedures to compare two or more recording systems make use of a comparison to a gold standard (i.e., gel electrodes) through two possible approaches, either simultaneously or serially (Ruffini et al., 2008). Most encountered parameters for evaluating EEG signal quality are comparisons of the power spectral densities, analysis of signal-to-noise ratio (SNR), and test of electrodes during applications (Tǎutan et al., 2014).

#### Signal Quality Analysis

In this work, the two systems were used for a simultaneous recording of EEG signals from contiguous scalp positions. Since our final aim is to use the proposed system for passive-BCI applications, for which spectral features will be used, the two systems have been compared in terms of variation of the impedance values over time and power spectra correlation.

Gel and water-based electrode impedance were considered good enough, for values respectively below 40 kΩ (Ferree et al., 2001) and 100 kΩ (Kappenman and Luck, 2010). Since the acceptability thresholds for the starting impedance values were set at two different values according to the literature, the impedances were not compared between the two systems for the same time point. However, to analyze the stability of each system during the recording, the impedances of the analyzed systems were evaluated at the beginning and the end of each experimental condition, and then, were linearly interpolated to have an impedance value every 10-min during a 40-min interval. Similarly, the effects related to the use of an unoptimized structure as sensor holders have been analyzed. Leveraging the time spent by the subject for task familiarization, they were asked to wear the Mindtooth Touch headset and, also in this case, the impedances were evaluated at the beginning and the end of each sample condition, and then, were linearly interpolated to have an impedance value every 10 min during a 40-min interval. It is noteworthy to highlight that in this case there is a temporal delay in impedance assessment because they have been measured in two different moments. The Wilcoxon signed-rank test (Bonferroni corrected) was performed to test the difference of each electrode concerning the starting point.

As impedance values affect SNR, we compared the number and the distribution of artifacts of the two systems. The Wilcoxon signed-rank test was performed to test the difference between the two systems in terms of the number of artifacts.

Finally, the power spectral densities (PSD) obtained through the two systems during open eyes conditions for each channel have been compared in terms of correlation. The higher the correlations between the frequency spectra of the gel and water electrode, the higher the possibility that both systems are equally capable of measuring spontaneous EEG (Zander et al., 2011). The open-eyes condition has been chosen because the EEG signal can be considered stationary during this condition. For each subject, Pearson's correlation has been computed between each couple of contiguous channels (e.g., AF3-water and AF3-gel) and for each band, that are Theta (3÷7 Hz), Alpha (7÷13), Beta (13÷26), and High Beta (21÷26). Each correlation value was Bonferroni corrected for multiple comparisons, therefore the threshold for significance has been set to α = 0.0025 (corresponding to a correlation value of R = 0.423). The obtained values have been statistically compared with the values of correlation obtained correlating the “Control” electrode and the AFz-gel electrode through a Wilcoxon signed-rank test (Bonferroni corrected). As they are both gel electrodes 1.5 cm apart, the obtained correlation can be considered the maximum achievable and the difference in correlation was only due to the distance between them.

#### Usability

To evaluate the usability and the comfort of the new system, participants were asked to answer specific questions regarding these aspects. In particular, the usability questions investigated the easiness of putting on and taking off the Mindtooth Touch headset. Participants could choose among four answers: Easy, Acceptable, Hard, Impossible. To assess the comfort, the participant had to choose among four possible answers: Comfortable, Acceptable, Tolerable, and Uncomfortable.

#### Neurometrics

For each human factor (workload, stress, and vigilance) a subset of channels and bands of interest have been chosen according to the literature and previous results (see Section Introduction for details). We defined “Neurometric” as the measure of each Human Factor, by using a specific combination of Global Field Power values (Di Flumeri et al., 2018). The Global Field Power (GFP) was calculated using the clean EEG for each frequency band of interest. The bands were defined accordingly with IAF values. Consequently, the following EEG bands were defined (Klimesch, 1999):

- Theta = (IAF – 6): (IAF – 2) Hz
- Alpha = (IAF – 2): (IAF + 2) Hz
- Beta = (IAF + 2): (IAF + 16) Hz
- Beta High = (IAF + 11): (IAF + 16) Hz

The workload neurometric is defined as the ratio between frontal activity in theta and parietal activity in the alpha band (Borghini et al., 2013). The theta rhythm increases, especially over the pre-frontal cortex, when the mental workload increases, while the alpha rhythm presents an inverse correlation with the mental workload, especially over the parietal cortex (Gevins et al., 1997). Therefore, the workload neurometric in this work was defined according to the literature as:


$$\begin{array}{l} Workload\;=\frac{Theta(AF8,AF7,AFz,AF3,AF4)}{Alpha(P3,P4,Pz)} \end{array}$$

Regarding stress measurement, the literature proved that there is a correlation between cortisol and brain activity in beta (Seo and Lee, 2010). A stress neurometric based on parietal brain activity in the high beta band was effectively tested during laboratory multitasking and was validated during realistic driving, proving that it is possible to have reliable stress measurements using the following (Sciaraffa et al., 2022):


$$\begin{array}{l} Stress=BetaHigh\;\left(P3,P4\right) \end{array}$$

From the neurophysiological point of view, vigilance-related processes involve mainly the right inferior frontal brain regions (Di Flumeri et al., 2019b; Sebastiani et al., 2020; Sciaraffa et al., 2021). Increased frontal activity in the beta band, more in right than in the left hemisphere, is correlated with vigilance decrement (Molina et al., 2013). Therefore, the vigilance neurometric was defined as:


$$\begin{array}{l} Vigilance=-Beta(AF4,AF8) \end{array}$$

The effectiveness of each neurometric (obtained by means of the water-based and the gel-based electrodes) was tested by statistically comparing through the Wilcoxon signed-rank test, their efficiency in discriminating experimental conditions. In particular, the following are compared for both laboratory and realistic tasks:

- workload neurometric was compared between easy and hard conditions;
- stress neurometric was compared between low and high-stress conditions;
- vigilance neurometric was compared between high and low vigilance conditions.

#### Machine Learning Model Calibration

Once obtained the neurophysiological information characterizing each mental state of interest, a machine learning-based approach has been employed to classify the two levels of each mental state (easy vs. hard for workload, and low vs. high for stress and vigilance, respectively) even at different time resolutions. The GFP values were used in this case as features:

- workload: AF7, AF8, AF3, AF4, and AFz in the Theta band and P3, P4, and Pz in the Alpha band (8 Features);
- stress: P3 and P4 in Beta High band (2 Features);
- vigilance: AF4 and AF8 computed in the Beta band (2 Features).

Each observation belonging to a specific run was labeled according to the level of mental state expected for that run. In the case where the classes (e.g., easy and hard) were too unbalanced because of the number of rejected artifacts, the Adaptive Synthetic (ADASYN) method was used to provide synthetic resampling (He et al., 2008). Once the observations for each class were balanced, and intra-subject approach was used to train the machine learning model (i.e., both training and testing were performed using the observations coming from the same subject). At this point two different procedures were performed, depending on the number of repetitions available for each task:

1. For each task having two repetitions per condition (i.e., the workload in a realistic setting, vigilance, and stress in both laboratory and realistic setting) one repetition has been used to train the algorithm and optimize the parameters, and one to test the algorithm.
2. For each task that does not have two repetitions per condition (i.e. workload in a laboratory setting), the k-fold cross validation has been performed (Schaffer, 1993). This allows to divide the dataset in k (k = 3) fold and to use two of them as a training dataset and one as a test dataset. Even in this case, all the possible combinations of training and testing sets have been analyzed.

Following preliminary analysis, the Random Forest was chosen as the model and the number of estimators and the max depth have been optimized in the range from 50 to 500, and from 1 to 50, respectively (Breiman, 2001).

The effectiveness of each model was assessed by computing the area under curve (AUC) (Bamber, 1975) between 0 and 1 at different temporal resolutions from 1 to 60 s. The whole described classification procedure has been repeated for water-based and gel-based systems and the two AUC curves obtained at different time resolutions have been statistically compared through Wilcoxon signed-rank test.

To test the generalizability of the proposed water-based passive BCI system, the same Random Forest model has been calibrated through a cross-task approach. For each human factor, the data recorded during the laboratory tasks (Study 1) has been used to calibrate the model. It has been then tested on the data recorded during the realistic driving (Study 2), and the AUC has been reported for time resolution from 1 to 60 s. This approach has been statistically compared with the intra-subject approach already described through Wilcoxon signed-rank test. Finally, each identified model was used to predict the probability of a specific observation belonging to a realistic session to have a high value of mental state (i.e., to belong to the high class). These curves were compared in terms of Pearson's correlation and distances between the curves using the root mean squared error (RMSE).

## Results

### Usability

**Figure 3:**
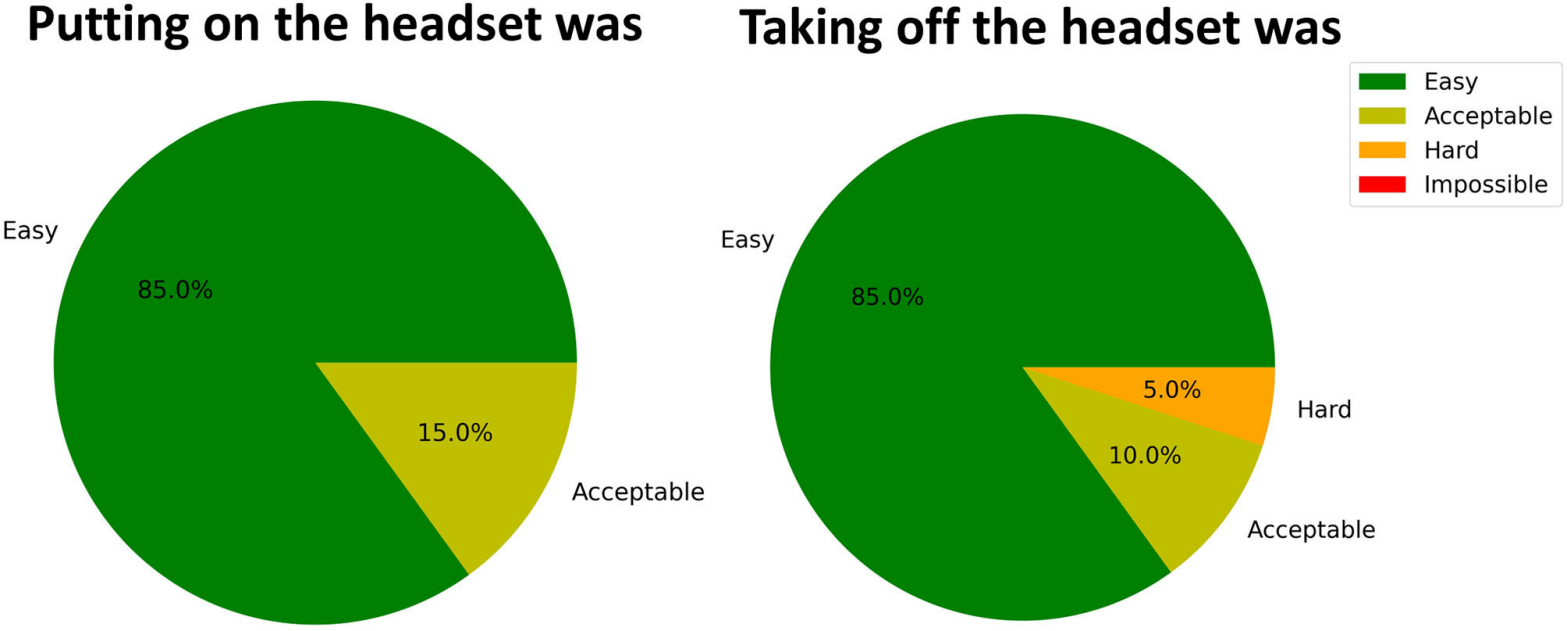
System usability. Easiness perception of headset put-on and take-off. The pie chart shows the percentage distribution of the perceived difficulty rated on the scale Easy, Acceptable, Hard, and Impossible.

For the usability assessment, the first aspect considered was referred to the easiness to wear the Mindtooth Touch headset. Putting on the headset was “Easy” for the 85% and “Acceptable” for the 15%. Moreover, we found that the time needed to put on the headset was 10 s on average (Figure 3). Therefore, participants were asked to take off the headset, and rate how it was. Eighty-five percent replied that it was “Easy,” 10% “Acceptable,” and 1 participant “Hard.” Regarding comfort, after 10 min of subjects' wearing the headset, 70% of them felt it was “Comfortable” and 30% felt it was “Acceptable”.

### Signal Quality

**Figure 4:**
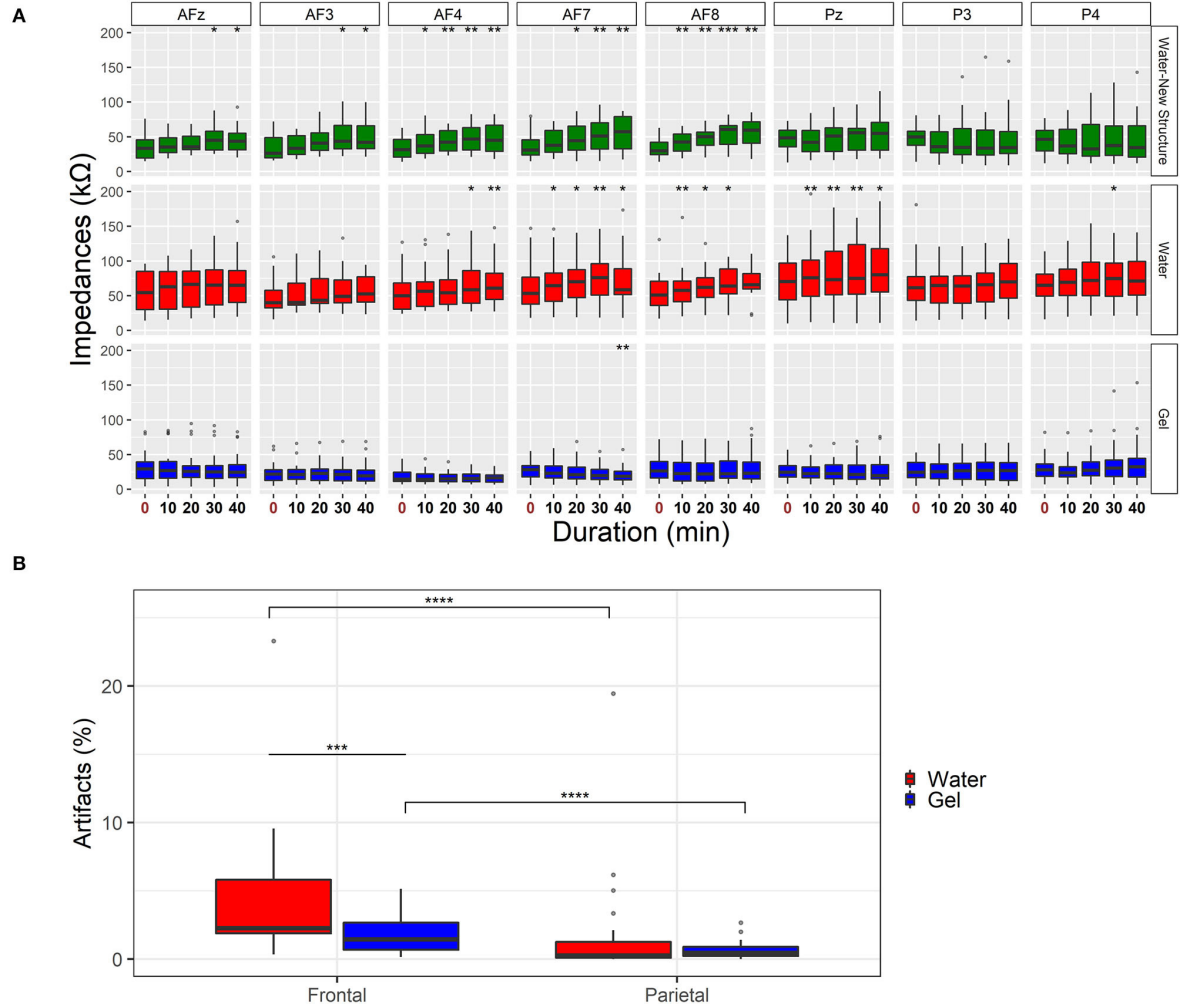
Analysis of impedances and percentage of artifacts. **(A)** Analysis of the stability of impedances over time for water-based (in red), gel (in blue), and water electrodes embedded on Mindtooth Touch headset. The asterisks show the results of the Wilcoxon signed-rank (Bonferroni corrected) test of the impedances of water and gel electrodes compared with the start point (*p < 0.05, **p < 0.01). **(B)** Percentage of artifacts in EEG signals. The boxplots show the distributions of the artifacts that occurred through the gel (blue) and water (red) acquisition system. Results have been obtained by averaging the electrodes in two groups: Frontal (AFz, AF3, AF4, AF7, and AF8) and Parietal (P3, P4, and Pz) channels. The asterisks show the results of the Wilcoxon signed-rank test (***p < 0.001, ****p < 0.0001).

Figure 4A shows that the water electrodes exhibited significant higher impedance values compared to starting point depending on the position and the duration of the recording. In particular, AF7 (*Z* = 26, *p* = 0.041), AF8 (*Z* = 26, *p* = 0.008), and Pz (*Z* = 18, *p* = 0.002) showed significantly higher impedances after 10 min of recording; AF4 (*Z* = 32, *p* = 0.019) and P4 (*Z* = 37, *p* = 0.038) after 30 min. The same electrodes embedded on the Mindtooth Touch Headset exhibited lower impedances even if all the frontal electrodes significantly increase over time starting from 30 min: AFz (*Z* = 30, *p* = 0.015), AF3 (*Z* = 34, *p* = 0.026), AF4 (*Z* = 17, *p* = 0.002), AF7 (*Z* = 19, *p* = 0.01), and AF8 (*Z* = 13, *p* = 0.0006). The gel electrodes did not show significant differences apart from the electrode AF7 after 40 min (*Z* = 142, *p* = 0.008).

The following analysis shows the number of artifacts for each recording technology (Figure 4B). The analysis shows a significantly higher number of artifacts by using the water compared to the gel sensors for frontal sites (*Z* = 198, *p* = 0.0001). Moreover, both the systems exhibited a higher number of artifacts on frontal sites compared to parietal sites (Water: *Z* = 210, *p* < 10^−5^; Gel: *Z* = 207, *p* < 10^−5^). Anyhow, the number of artifacts with water-based technology was about 5% on average.

**Figure 5:**
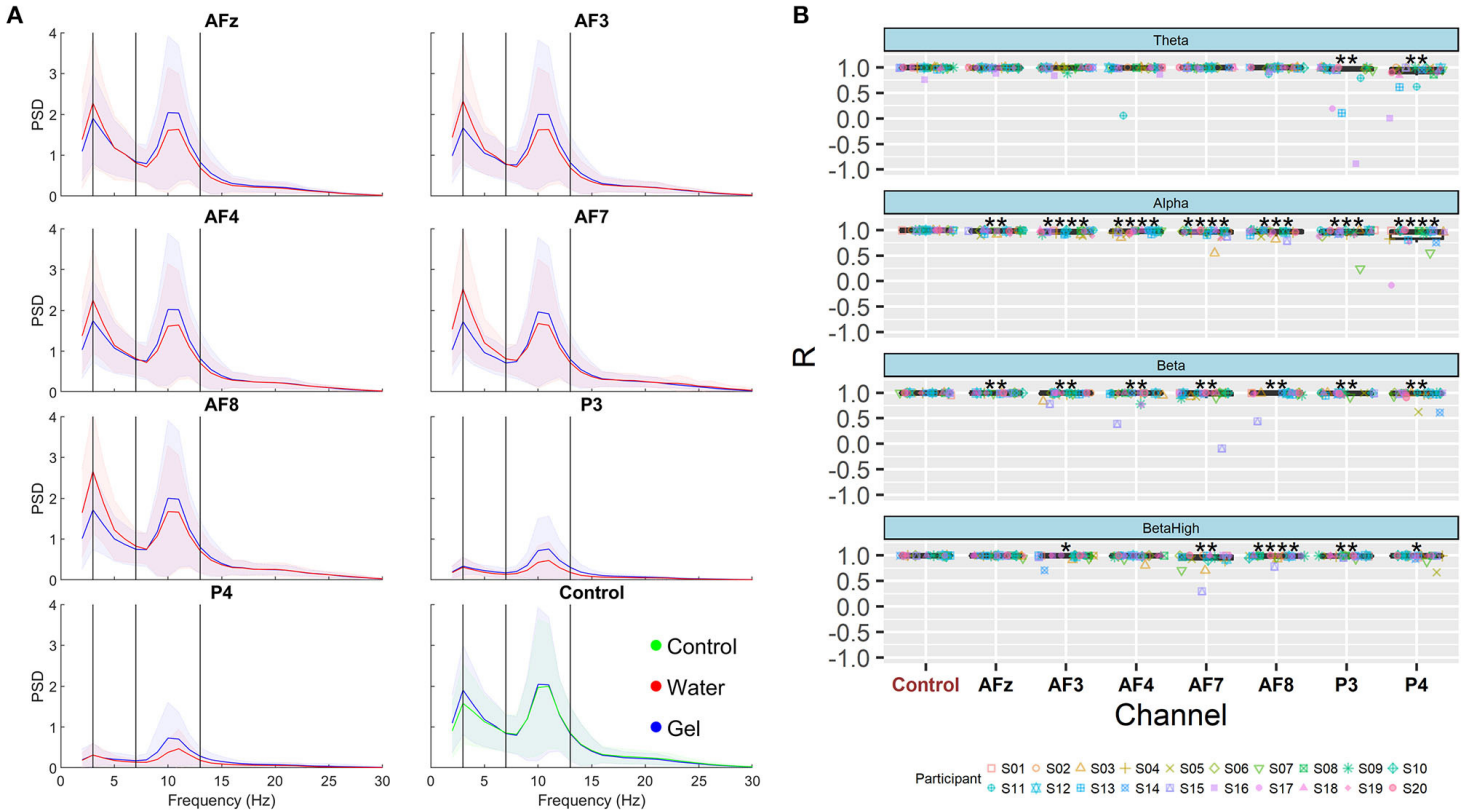
Spectral analysis. **(A)** EEG spectra for each channel in the interval 2–30 Hz. Spectra obtained with water electrodes are shown in red, and spectra obtained with gel electrodes are shown in blue. The last subplot shows the AFz-gel and control electrode. The solid lines represent the average of the spectra over the population, and the shadowed areas represent the standard deviation. **(B)** Boxplots of Pearson's correlation values computed between water and gel electrodes for each channel and band. Each distribution has been compared with the values obtained from AFz-control correlation. The asterisks show the results of the Wilcoxon signed-rank test (Bonferroni corrected, *p < 0.05, **p < 0.01, ***p < 0.001, ****p < 0.0001).

At this point, we investigated if the higher impedance values of the water technology negatively affect the spectral features used to compute EEG neurometrics related to the selected human factors. Figure 5A shows the averaged spectra obtained with both sensors after the artifacts' rejection. These spectra were analyzed in terms of Pearson's correlation (R). Figure 5B shows the distributions of the R values for each frequency band. Nine of the 720 obtained R values were lower than 0.423, therefore, not significant according to Bonferroni correction. The correlation is on average higher than 0.95 for each channel and band. These distributions were statistically compared with the R values obtained correlating the spectra of AFz-gel and control electrode. The Wilcoxon signed-rank test (Bonferroni corrected) shows that the correlation between gel and water spectra is significantly lower compared to the Control-AFz spectra especially for alpha and beta bands, whereas this is less evident in the theta band.

### Study 1: Test on Laboratory Tasks

**Figure 6:**
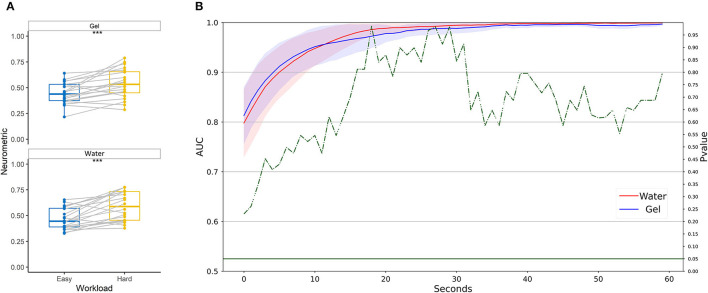
Workload discrimination between easy and hard conditions in laboratory settings. **(A)** Statistical comparison has been performed for water-based and gel-based Neurometrics. The asterisks show the results of the Wilcoxon signed-rank test (***p < 0.001). **(B)** AUC values obtained by water (red) and gel (blue) electrodes for different time resolutions (1–60 s). The solid lines represent the median of the AUC among the participants. The shadows represent the mean absolute deviation of the AUC across participants. The dashed green line represents the results in terms of *p*-values of the Wilcoxon signed-rank test. The threshold of significance (*p* = 0.05) has been highlighted in the same color.

During multitasking tasks, single and multitasking levels were considered as low and high workload conditions, respectively. Figure 6A shows that the Neurometrics of workload computed by using both the technologies showed a significant difference between the easy and the hard condition (Gel: *F* = 19, *p* = 0.0006; Water: *F* = 14, *p* = 0.0002). The machine learning-based analysis (Figure 6B) did not highlight any difference between the AUC values of the two systems, at any time resolution, showing for both the technologies AUC on average higher than 0.8.

**Figure 7:**
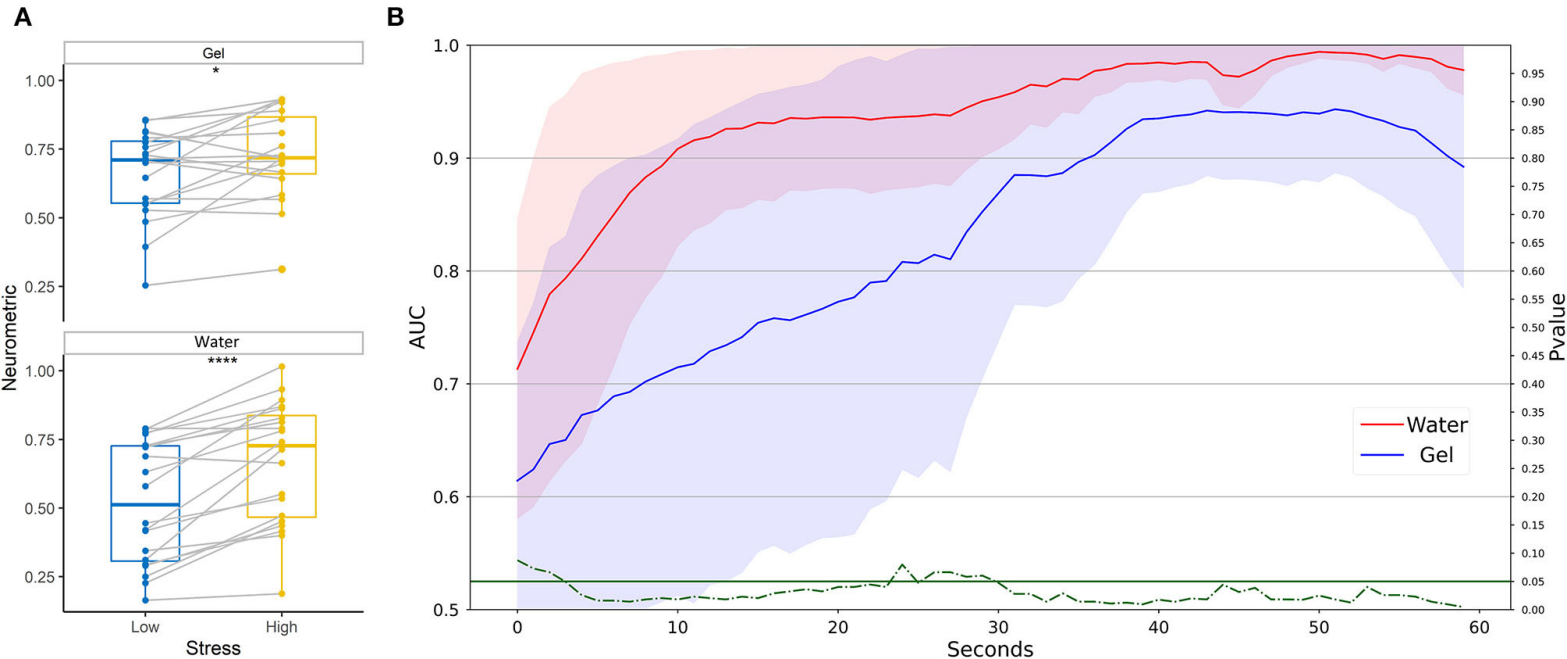
Stress discrimination between low and high conditions in laboratory settings. **(A)** Statistical comparison has been performed for water-based and gel-based Neurometrics. The asterisks show the results of the Wilcoxon signed-rank test (*p < 0.05, ****p < 0.0001). **(B)** AUC values obtained by water (in red) and gel (in blue) electrodes for different time resolutions (1–60 s). The solid lines represent the median of the AUC among the participants. The shadows represent the mean absolute deviation of the AUC across participants. The dashed green line represents the results in terms of *p*-values of the Wilcoxon signed-rank test. The threshold of significance (*p* = 0.05) has been highlighted in the same color.

Neurometrics of stress computed by using both technologies showed a significant difference between the low stress and the high-stress conditions (Gel: *Z* = 45, *p* = 0.024; Water: *Z* = 3, *p* < 10^−5^) (Figure 7A). The machine learning-based analysis (Figure 7B) highlighted a statistically significant difference between the AUC values of the two systems, starting from 4 s resolution. In particular, the water-based system provided significantly higher AUC than gel-based ones.

**Figure 8:**
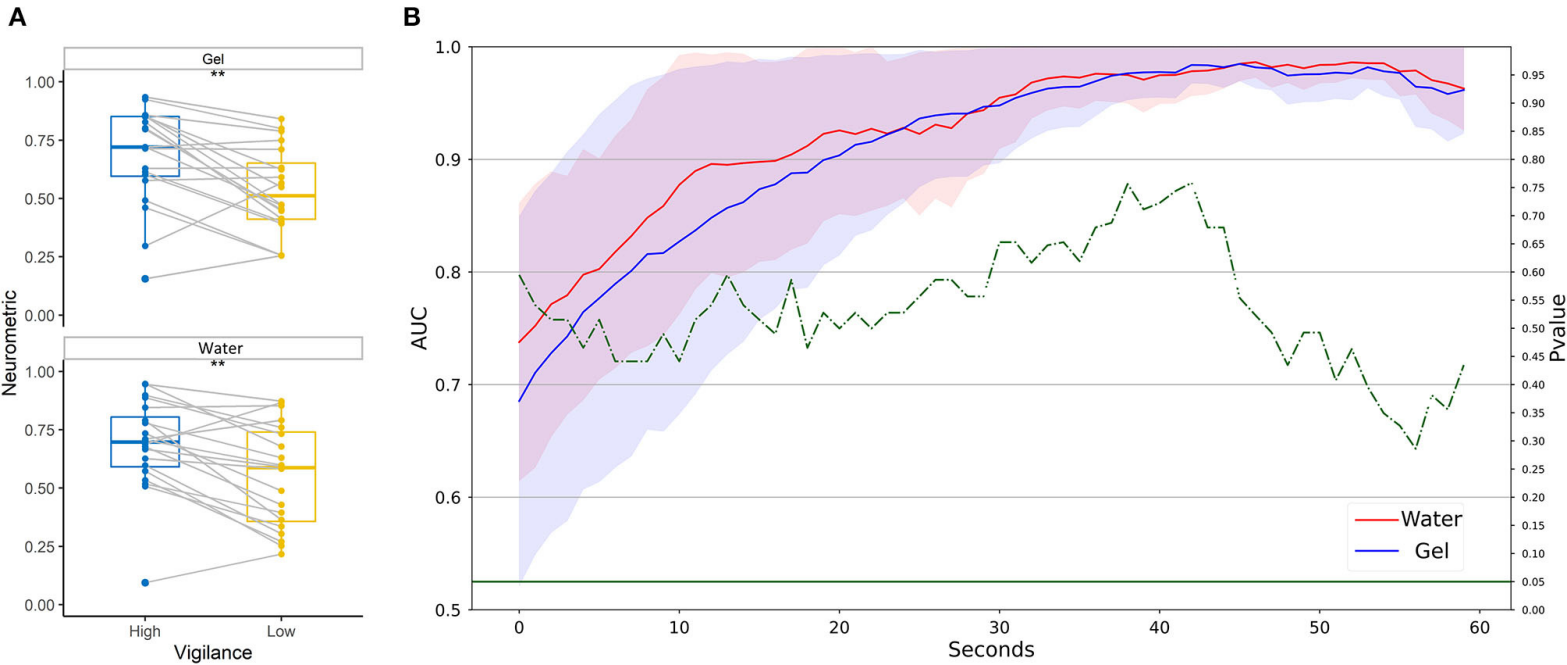
Vigilance discrimination between low and high conditions in laboratory settings. **(A)** Statistical comparison has been performed for water-based and gel-based Neurometrics. The asterisks show the results of the Wilcoxon signed-rank test (**p < 0.01). **(B)** AUC values obtained by water (in red) and gel (in blue) electrodes for different time resolutions (1–60 s). The solid lines represent the median of the AUC among the participants. The shadows represent the mean absolute deviation of the AUC across participants. The dashed green line represents the results in terms of *p*-values of the Wilcoxon signed-rank test. The threshold of significance (*p* = 0.05) has been highlighted in the same color.

Neurometrics of vigilance (Figure 8A) computed by using both the two technologies showed a significant difference between the High vigilance and the Low vigilance conditions (Gel: *Z* = 179, *p* = 0.0042; Water: *Z* = 183, *p* = 0.0023). The machine learning-based analysis (Figure 8B) did not highlight any difference between the AUC values of the two systems, at any time resolution. The AUC overcame 0.8 at 10 s of temporal resolution.

### Study 2: Test on Realistic Driving

**Figure 9:**
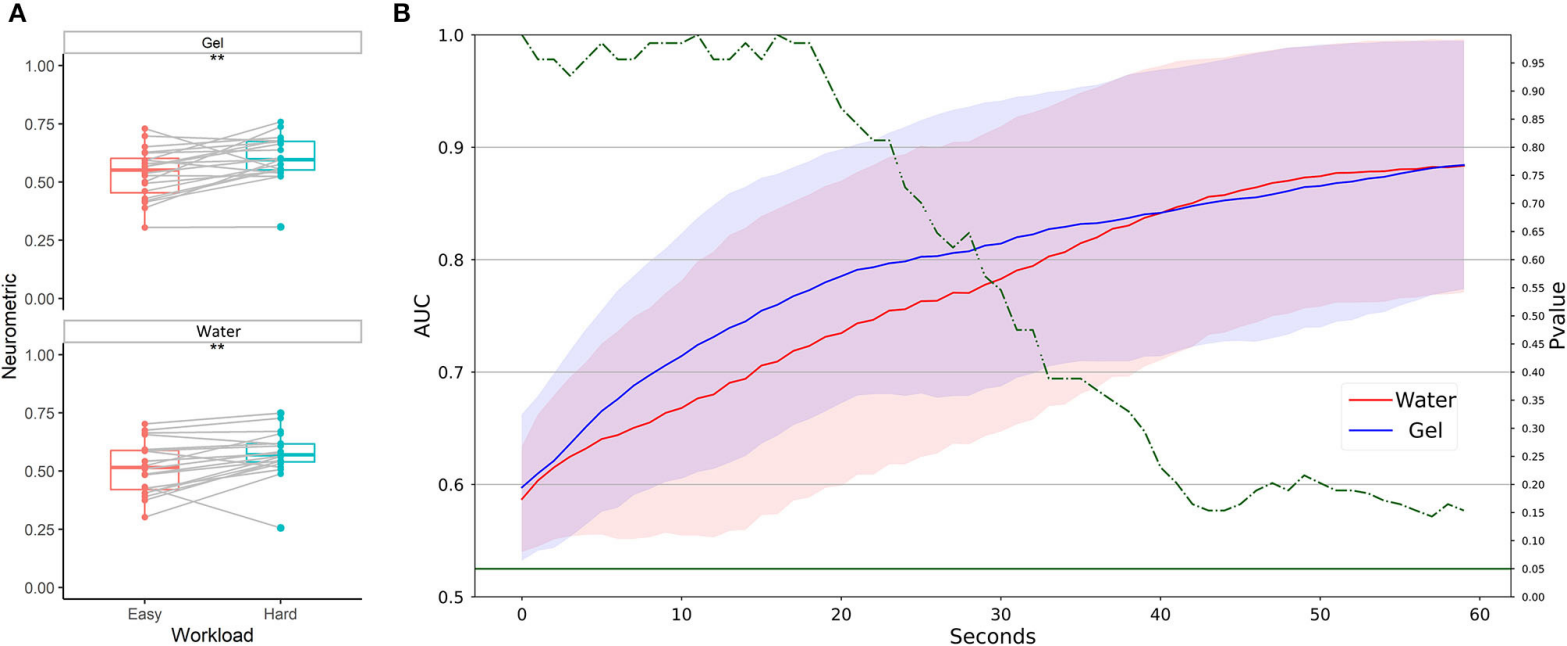
Workload discrimination between easy and hard conditions in a realistic setting. **(A)** Statistical comparison has been performed for water-based and gel-based Neurometrics. The asterisks show the results of the Wilcoxon signed-rank test (**p < 0.01). **(B)** AUC values obtained by water (in red) and gel (in blue) electrodes for different time resolutions (1–60 s). The solid lines represent the median of the AUC among the participants. The shadows represent the mean absolute deviation of the AUC across participants. The dashed green line represents the results in terms of *p*-values of the Wilcoxon signed-rank test. The threshold of significance (*p* = 0.05) has been highlighted in the same color.

Neurometrics of workload computed by using both the two technologies showed a significant difference between the easy and the hard condition (Gel: *Z* = 34, *p* = 0.0064; Water: *Z* = 35, *p* = 0.0073) (Figure 9A). The machine learning-based analysis (Figure 9B) did not highlight any difference between the AUC values of the two systems, at any time resolution. For both systems, the AUC overcomes 0.8 at 30 s of temporal resolution.

**Figure 10:**
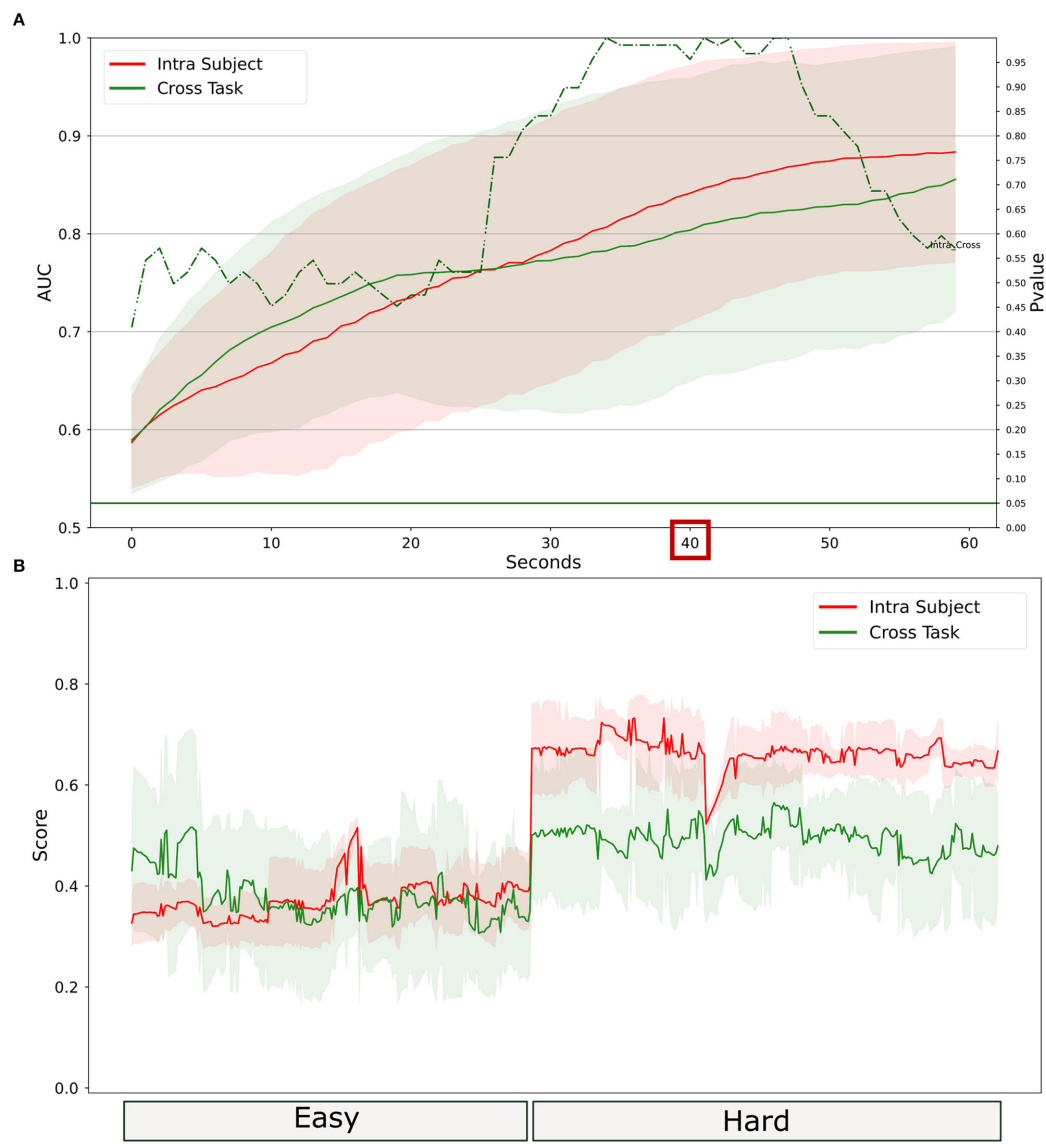
Comparison between intra-subject and cross-task calibration for workload discrimination in a realistic setting. **(A)** AUC values obtained by water electrodes through intra-subject (red) and cross-subject (green) calibration for different time resolutions (1–60 s). The solid lines represent the median of the AUC among the participants. The shadows represent the mean absolute deviation of the AUC across participants. The dashed green line represents the results in terms of *p*-values of the Wilcoxon signed-rank test. The threshold of significance (*p* = 0.05) has been highlighted in the same color. **(B)** The workload index obtained from each of the two models at 40 s of temporal resolution. The line represents the average over the population, the shadow represents the standard deviation.

To investigate the ability to generalize the proposed water-based passive-BCI system, the data recorded during the laboratory tasks have been used to calibrate the system. Therefore, it has been tested on simulated driving. Results in Figure 10A showed that the system reached an AUC value higher than 0.8 with 40 s of temporal resolution. The cross-task approach did not perform significantly differently compared to the intra-subject approach. In Figure 10B the related scores obtained at 40 s of temporal resolution indicate that there is a medium correlation between the outputs of the two models. The median over the population of the correlation between the scores obtained is R = 0.60 (IQR = 0.54, all *p* < 10^−5^), and the RMSE = 0.16 (IQR = 0.05).

**Figure 11:**
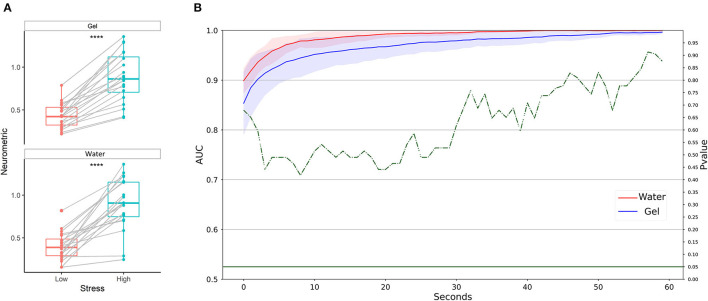
Stress discrimination between low and high conditions in a realistic setting. **(A)** Statistical comparison has been performed for water-based and gel-based Neurometrics. The asterisks show the results of the Wilcoxon signed-rank test (****p < 10^−5^). **(B)** AUC values obtained by water (in red) and gel (in blue) electrodes for different time resolutions (1–60 s). The solid lines represent the median of the AUC among the participants. The shadows represent the mean absolute deviation of the AUC across participants. The dashed green line represents the results in terms of *p*-values of the Wilcoxon signed-rank test. The threshold of significance (*p* = 0.05) has been highlighted in the same color.

Neurometrics of stress (Figure 11A) computed by using both the two technologies showed a significant difference between the low stress and the high-stress conditions (Gel: *Z* = 0, *p* < 10^−5^; Water: *Z* = 0, *p* < 10^−5^). The machine learning-based analysis (Figure 11B) did not highlight any difference between the AUC values of the two systems, at any time resolution, showing for both the technologies AUC higher than 0.8 on average.

**Figure 12:**
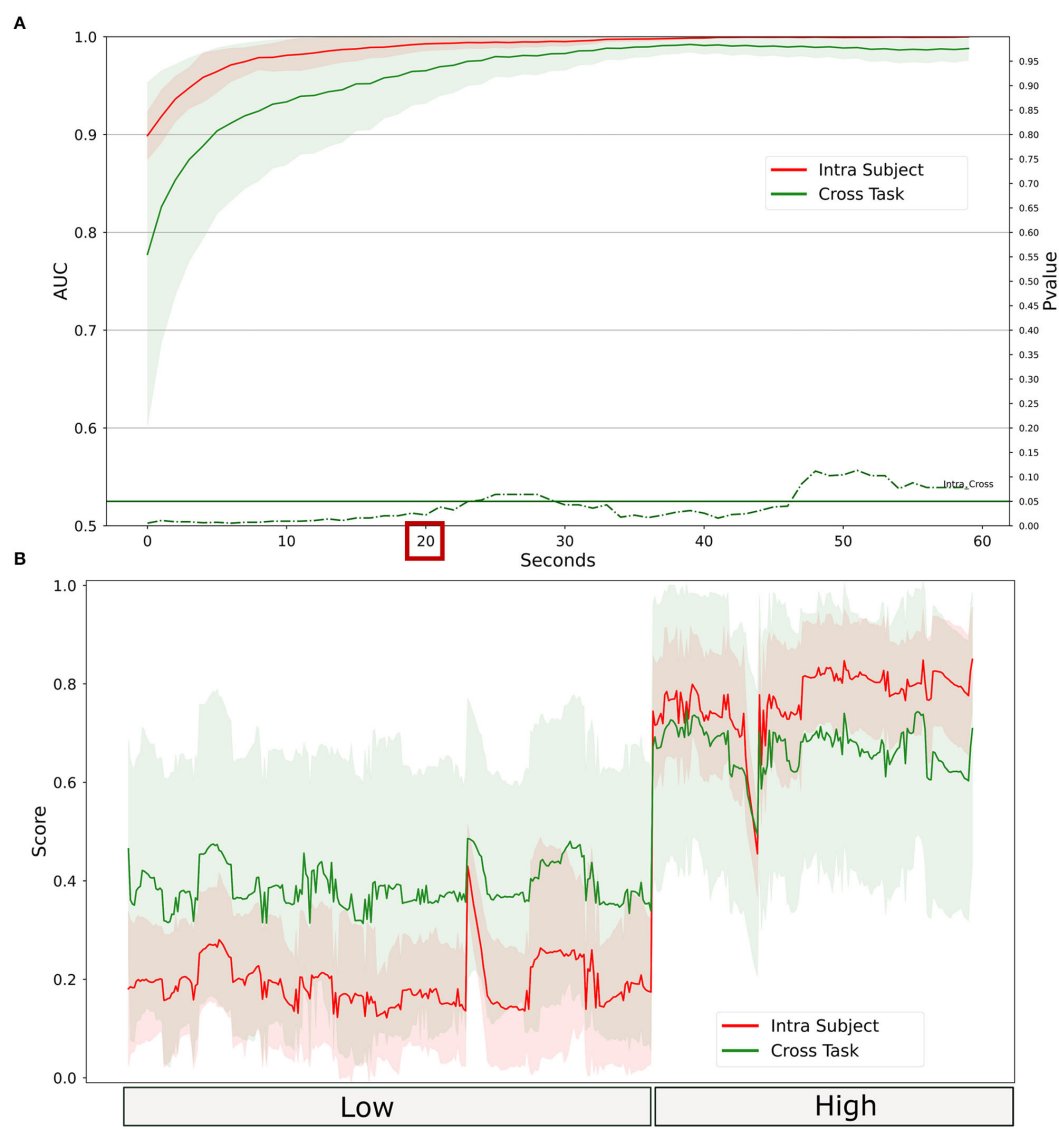
Comparison between intra-subject and cross-task calibration for stress discrimination in a realistic setting. **(A)** AUC values obtained by water electrodes through intra-subject (red) and cross-subject (green) calibration for different time resolutions (1–60 s). The solid lines represent the median of the AUC among the participants. The shadows represent the mean absolute deviation of the AUC across participants. The dashed green line represents the results in terms of *p*-values of the Wilcoxon signed-rank test. The threshold of significance (*p* = 0.05) has been highlighted in the same color. **(B)** The stress index obtained from each of the two models at 20 s of temporal resolution. The line represents the average over the population, and the shadow represents the standard deviation.

Even for the stress, it has been investigated the ability to generalize of the proposed water-based passive-BCI system. First, the data recorded during the laboratory multitasking have been used to calibrate the system. Then, the calibrated system has been tested on the simulated driving. Results in Figure 12A showed that the system reached AUC values higher than 0.8 almost in real time. The intra-subject calibration performed significantly better than the cross-task approach until a temporal resolution of 45 s. The related scores shown in Figure 12B indicated that there is a high correlation between the outputs of the two models. The median (IQR) over the population of the correlation between the scores obtained is R = 0.89 (IQR = 0.28, all *p* < 10^−3^), and the RMSE = 0.27 (0.16).

**Figure 13:**
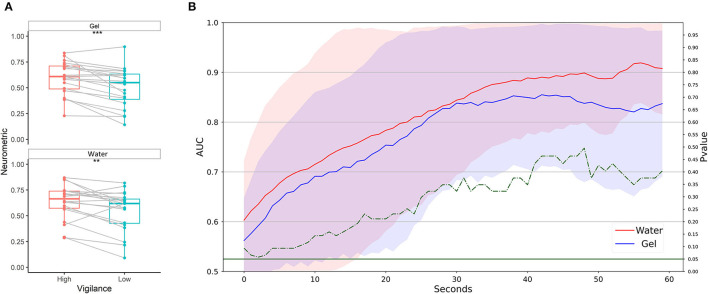
Vigilance discrimination between low and high conditions in realistic settings. **(A)** Statistical comparison has been performed for water-based and gel-based Neurometrics. The asterisks show the results of the Wilcoxon signed-rank test (**p < 0.01, ***p < 0.001). **(B)** AUC values obtained by water (in red) and gel (in blue) electrodes for different time resolutions (1–60 s). The solid lines represent the median of the AUC among the participants. The shadows represent the mean absolute deviation of the AUC across participants. The dashed green line represents the results in terms of *p*-values of the Wilcoxon signed-rank test. The threshold of significance (*p* = 0.05) has been highlighted in the same color.

Neurometrics of vigilance (Figure 13A) computed by using the two technologies showed a significant difference between the High vigilance and the Low vigilance conditions (Gel: *Z* = 198, *p* = 0.00013; Water: *Z* = 177, *p* = 0.0056). The machine learning-based analysis (Figure 13B) did not highlight any difference between the AUC values of the two systems, at any time resolution. AUC values higher than 0.8 were reached at 30 s of temporal resolution.

**Figure 14:**
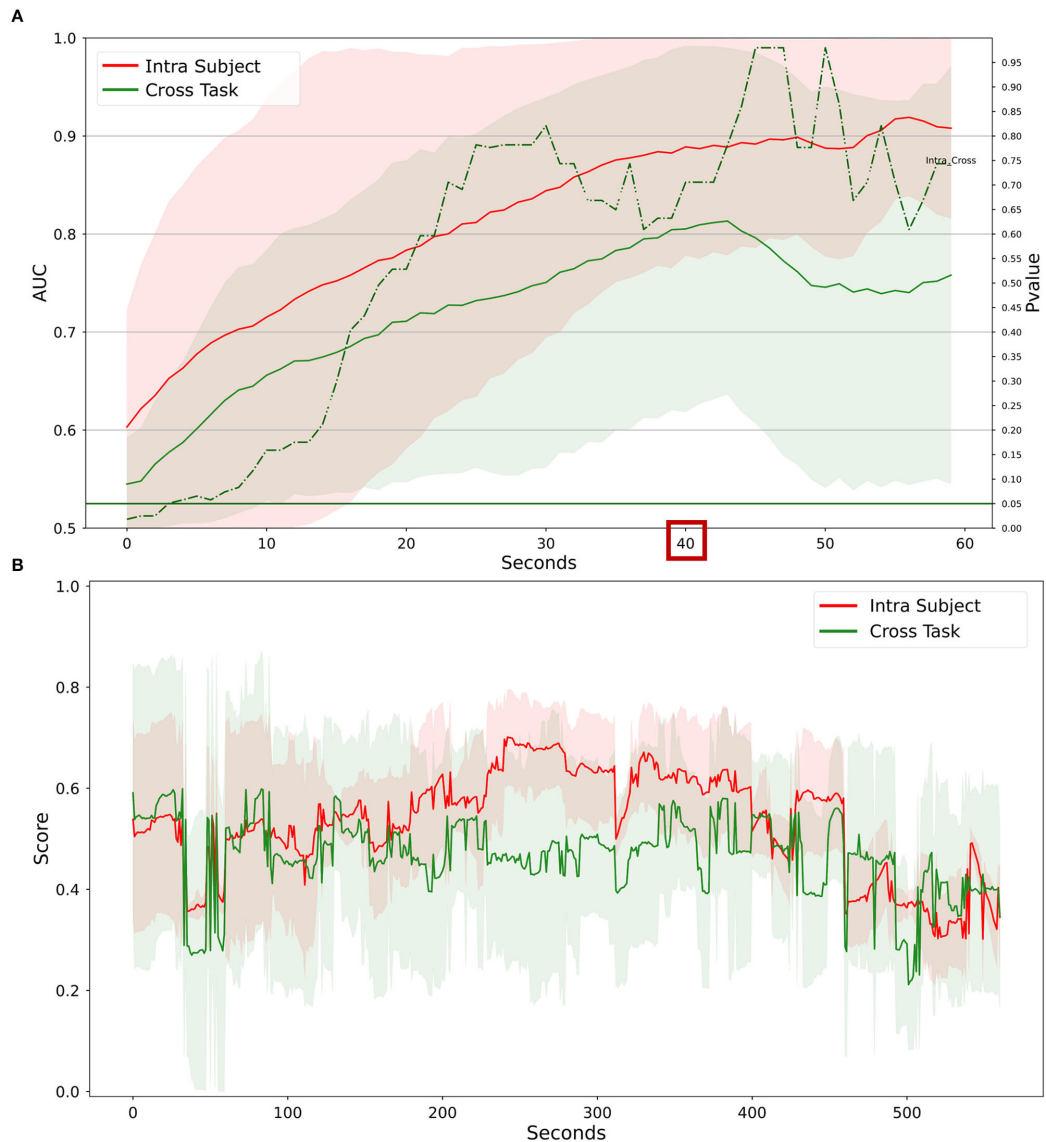
Comparison between intra-subject and cross-task calibration for vigilance discrimination in a realistic setting. **(A)** AUC values obtained by water electrodes through intra-subject (red) and cross-subject (green) calibration for different time resolutions (1–60 s). The solid lines represent the median of the AUC among the participants. The shadows represent the mean absolute deviation of the AUC across participants. The dashed green line represents the results in terms of *p*-values of the Wilcoxon signed-rank test. The threshold of significance (*p* = 0.05) has been highlighted in the same color. **(B)** The vigilance index obtained from each of the two models at 40 s of temporal resolution. The line represents the average over the population, the shadow represents the standard deviation.

The data recorded during the PVT has been used to calibrate the water-based passive-BCI system. Then, the calibrated system has been tested on the simulated driving. Results in Figure 14A showed that the cross-task and the intra-subject calibration did not provide significantly different performances, even if the intra-subject approach provided the highest values of AUC overcoming 0.9. The scores shown in Figure 14B have been analyzed in terms of correlation and distances between the curves. The median over the population of the correlation between the scores obtained is R = 0.48 (IQR = 0.36, all *p* < 0.01), and the RMSE = 0.19 (0.12).

## Discussion

Passive-BCI systems are an effective tool for strengthening human-machine interaction. The use of such technologies has been proved to be particularly useful in those human-centered areas where safety and adaptive training are relevant concerns. However, their employment away from laboratories or research activities has not yet taken place. The reason for this lies in shortcomings in three fundamental aspects: reliability, generalizability, and ease of use. In this context, this work aimed to evaluate a new water-based passive-BCI system covering (i) each of the aforementioned features; (ii) laboratory and realistic settings; and (iii) three different human factors.

### Water-Based vs. Gel-Based System: Impact on the Ease of Use and Reliability

The results obtained showed that the use of the new water-based system represents a decisive improvement in terms of usability and that the signal quality guarantees result comparable to those obtained with the gel system.

The usability test of the Mindtooth Touch headset embedded with water-based electrodes showed that it allows a quick and easy application, 10 s on average, avoiding every kind of damaging skin preparation. However, one of the participants found it “Hard” to take off the headset (Figure 3). This issue was mainly due to the imperfections at the corners of the electrode holders, which could get stuck in the hair. This possible issue will be mitigated by smoothing and rounding edges. No participants found it uncomfortable to wear the headset. After 10 min, 30% considered wearing the headset acceptable. By interviewing the participants, we understood that the comfort issue was mainly due to the pressure induced by the frontal holders. One solution implemented to mitigate this issue was to place a soft foam pad attached with Velcro to the front to evenly distribute pressure over the front head and improve overall comfort. In general, due to the ease of use and comfort, the system proved to be ready for recording EEG out of the lab, and its use will be validated in real settings like pilots and driver training and during working activities.

To analyze the stability of each system during the recording, the impedances of the analyzed systems were evaluated at the beginning and the end of each experimental condition. The results demonstrated that the impedances of gel electrodes did not significantly vary over time, except for AF7 showed a significant reduction compared to the start of the experiment (Figure 4A). This is because the impedances of gel electrodes are usually subjected to a natural adaptation before the gel gets dry (Toyama et al., 2012). The impedances of water electrodes are on average lower than 100 kΩ. Unlike the parietal electrodes, the frontal electrodes showed a significant increment of impedances in the 40-minute window that has been analyzed. This indicates that the electrodes on the forehead were subjected to a faster drying than the parietal, whereas the presence of hair can prevent this from happening. However, these differences did not depend only on the electrochemical characteristics of the electrodes, but also on the mechanical fixation. Analyzing the same water electrodes placed on the specially designed structure, the impedances decreased to 50 kΩ, even if they showed the same increase on the frontal sites over time. As seen so far, the Mindtooth Touch structure in purpose has been specifically designed to facilitate the use of water electrodes while promoting comfort, time spent fitting the headset, and ease of use. However, as the recording advances, and the saline solution evaporates, impedances increase over time and this can negatively affect the data quality of long recordings (Noreika et al., 2020). For long recording (more than 30 min) the saline solution could be reapplied using a pipette and without removing the headset.

Higher impedances characterizing the water-based system could impact on signal to noise ratio (SNR) of EEG signals because of the common-mode rejection and cephalic skin potentials. Common mode rejection refers to the ability of a recording system to reject noise that is in common with the recording sensors and reference electrodes. As the electrode impedance increases, the common-mode rejection of the system decreases, and the SNR of the recording decreases as well. To deal with the problem of decreased common-mode rejection with high electrode impedances, amplifiers with a higher input impedance have been used. In this regard, the used LiveAmp amplifier has a very high input impedance (i.e., 200 MΩ), so this first problem could be automatically solved. Electrode impedances that are too high may lead to a second problem that cannot be solved using changes to the amplifier's input impedance, namely, an increase in skin potential artifacts. Skin potentials arise because of the standing electrical potential that is normally present between the inside and the outside of the skin (Edelberg, 1972). Current literature demonstrated that high impedance values (i.e., 200 kΩ) could increase noise and induce changes in a frequency lower than 5 Hz since skin potentials induce slow voltage variations (Ferree et al., 2001; Kappenman and Luck, 2010). In this regard, both systems showed a higher number of artifacts on frontal electrodes compared to parietal ones (Figure 4B). Frontal electrodes seem more sensitive to unrelated electrical signals originating from non-brain physiological activities in particular muscular and ocular activities, reflecting a non-homogenous distribution of artifacts over the scalp (Abdi-Sargezeh et al., 2021). Even if both systems showed a percentage of artifacts on average that is lower than 5% of the total amount of data recorded, the frontal water electrodes recorded a significantly higher number of artifacts compared to gel ones. In contrast, the number of artifacts on parietal sites is not different between the two systems. The higher number of artifacts on frontal sites could be due to the mechanical fixation of the used EEG cap that disadvantaged the water electrodes. As demonstrated, water sensors placed on the Mindtooth Touch structure guarantee better contact and lower impedances.

From the spectral perspective, we investigated if the higher impedance values of the water-based technology compared to the gel-based technology would negatively affect the spectral features used to compute EEG neurometrics. Visual inspection of the spectra obtained through water and gel electrodes showed that the shape is very similar (Figure 5A). The most evident difference is that water electrodes showed higher values in the theta band, while gel showed higher values in the alpha band. The correlation analysis of specific EEG features (e.g., theta, alpha, beta, and high beta bands) during open eyes conditions suggested that the correlation between gel and water features is high (R > 0.95). Comparing the distribution of R values with that obtained from correlating the AFz-gel electrode with the Control electrode (correlation between two gel electrodes was used as a benchmark, therefore eventually differences in correlation depend on the distances between the two electrodes), we found that even if the correlation between gel and water spectral features is high, it is significantly lower compared to the control-AFz spectra especially for alpha and beta bands, whereas this is less evident in the theta band. These differences found between the different bands are coherent with similar spectral comparisons performed between a gel-based and water-based system (Topor et al., 2021). The authors found that the water-based system behaves significantly different from the gel-based in the detection of high-beta desynchronization, that the theta band was the less consistent between the two systems, and that there was a frontal shift of maximal power in theta and alpha bands. A slow drift noise has been hypothesized to be the cause of this. This correlation analysis has been performed on a highly controlled condition (i.e., open eyes), however, the degree of freedom during the analyzed paradigms could impact the spectral correlation depending on the type of electrode (Tautan et al., 2014). The possible loss of quality of EEG signal recorded from water-based electrodes could not impact neurometrics assessment when the experimental task is very controlled (i.e., the participant does not move too much, simple tasks, low number of expected artifacts), but it could affect the computation in real settings (i.e., higher number of expected artifacts, movement of the head, and real tasks). For this reason, the employment of the proposed water-based passive BCI has been tested in both laboratory and realistic settings.

#### Study 1: Human Factors Discrimination During Laboratory Tasks

The aim of Study 1 is to test the proposed system during controlled laboratory tasks and to use these data to calibrate the passive-BCI system based on a machine learning model. Before going on to test a system during a realistic application, the step to a more controlled application is a must in the case of passive-BCI. One of the reasons is that the targeted human factors are not independent variables. For example, stress is affected by workload level, conversely, the effort involved in coping with stress actually adds to the task demands (Stanton and Young, 2000); a variation in workload is associated with a variation in fatigue (Roy et al., 2016), and so on. Thanks to controlled tasks performed in the laboratory it is more likely to induce a variation of a single human factor per time compared to realistic contexts. Therefore, two standard tasks have been used to elicit workload, stress, and vigilance variations.

Multitasking has been chosen as a laboratory task to induce workload and stress modulation. This task is similar to the simultaneous capacity (SIMKAP) task usually used to elicit workload variation and whose effectiveness in eliciting stress variation has been already proved (Wetherell and Sidgreaves, 2005; Lim et al., 2018). Multitasking has been preferred to operate-orientated task paradigms like the multi-attribute task battery (MATB) because it consists of common tasks like entry numbers, mathematical additions, bar filling, and auditory test, and the time of familiarization is shorter than those required for the MATB (Comstock, 1992). Compared to multitasking, the MATB was born in the context of aviation research and usually requires 1 week of training to avoid any learning bias. As the employed task should be used to calibrate a cross-task system to be used in a realistic environment, it is necessary that the training is fast and straightforward.

The results obtained on multitasking showed that the neurometrics obtained with both gel and water electrodes were able to significantly discriminate the high level of the workload from the low. This confirms previous research, where a measure of workload based on frontal activity in the theta band and parietal activity in the alpha band has been consolidated and applied in different contexts (Borghini et al., 2014). The same neurophysiological information used as features to calibrate a machine learning model based on a random forest showed AUC values higher than 0.8 for high temporal resolution, and values of almost 1 for resolution lower than 30 s in the laboratory setting. There is not a statistically significant difference between the performance obtained with the two systems. On a similar multitasking dataset, 69.2% of accuracy has been reached through a support vector machine (Lim et al., 2018) and 82.57% accuracy using the LSTM model (Chakladar et al., 2020).

Analogously, the neurometrics obtained with gel and water electrodes were both able to significantly discriminate between high and low stress during the multitasking levels. Moreover, the discrimination of stress levels during the laboratory task through the machine learning-based approach showed significantly different performances between the water and the gel systems. In particular, the AUC values obtained with the water-based system are significantly higher than those obtained with the gel ones by about 10%. The proposed passive BCI system employed to discriminate between two levels of stress showed higher performance compared with the gold standard during the laboratory experiment. We could hypothesize that this increment could be associated with spectral differences discovered during spectral analysis. Comparing these outcomes with the literature, Attallah (2020) reached an accuracy of 99.26% for discriminating between two levels of stress with a KNN model.

Finally, both systems provided neurometrics that can significantly discriminate the high level of vigilance from the low during the PVT. The discrimination of the two levels of vigilance during the PVT, through the machine learning-based approach provided AUC values higher than 0.7 on average for high temporal resolution, and values higher than 0.95 for resolution lower than 30 s in the laboratory setting. There is not a statistically significant difference between the performance obtained with the two systems. Therefore, the proposed passive BCI system employed to discriminate between two levels of vigilance did not behave differently from the gold standard. The results obtained are in line with the literature: using an SVM model, Mehreen et al. (2019) obtained 76% of accuracy, while Wei et al. (2018) reached 0.85 of AUC using a non-hair bearing EEG. In Choi et al. (2019), the PVT task has been used to effectively train a system to detect instantaneous drowsiness with EEG. Using an extreme gradient boosting as a machine learning classifier, the authors tested both a wired/wet EEG and a wireless/dry EEG verifying that the latter reached 0.81 of AUC, 7 % less than the standard system.

In conclusion, analyzing the results of Study 1 the proposed system did not behave differently from the gel-based system when neurometrics are used to discriminate between human factors levels. Moreover, water-based electrodes overcame the traditional ones for the discrimination of the two levels of stress and did not behave significantly differently in case of vigilance or workload discrimination.

#### Study 2: Human Factors Discrimination During Realistic Driving

The neurometrics obtained with both gel and water electrodes were able to significantly discriminate the high level of the workload from the low during realistic driving. Analogously, the workload during the driving task has been discriminated with AUC around 0.6 for high resolution (i.e., 1 s) and the threshold of 0.8 was surpassed only after 30 s of time resolution. Also, in this case, there was no significant difference between the two systems. Therefore, the proposed passive BCI system employed to discriminate between two levels of workload showed higher performance in laboratory settings compared to the realistic ones for the same time resolution. However, it did not behave differently from the gold standard. Comparing the obtained results with the literature, 72% accuracy has been obtained through a support vector machine and a driving simulator (Hernández et al., 2018). The author (Fan et al., 2015) obtained an accuracy of 81.46% using a KNN and a driving system in virtual reality.

The neurometrics obtained with gel and water electrodes were both able to significantly discriminate between high and low stress in realistic settings. Moreover, there is not a statistically significant difference between the performance obtained with the two machine -learning based systems during the realistic driving. Both systems showed an AUC higher than 0.9 for a temporal resolution that is lower than 4 s. Therefore, the proposed passive BCI system employed to discriminate between two levels of stress, showed higher performance compared with the gold standard during the laboratory experiment, while they behave similarly during realistic tasks. We could hypothesize that this minimal decrease could be associated with the differences in the position of the electrodes, thus, representing one limitation of the current study. In Halim and Rehan (2020) more than 50 automotive drivers have been tested during various driving situations. An SVM model reached 97.95% of accuracy in discriminating rest and stress during driving through EEG signals.

The neurometrics obtained with gel and water electrodes were both able to significantly discriminate the high level of vigilance from the low ones in realistic settings. The vigilance during the driving task has been discriminated with AUC around 0.6 for high temporal resolution (i.e., 1 s) and the water-based electrodes allowed to overcome 0.9 on average AUC for temporal resolution lower than 50 s. Therefore, the proposed passive BCI system that was employed to discriminate between two levels of vigilance showed higher performance in a laboratory setting compared to the real ones for the same time resolution. However, it did not behave differently from the gold standard. Similar results have been already obtained through dry electrodes in the driving context. A case study involving 15 participants in an immersive virtual driving environment demonstrates the reliability and the feasibility of predicting the driver's vigilance through support vector regression (Lin et al., 2014).

### Generalizability of the Cross-Task Approach

The generalizability of a passive-BCI system depends strictly on the implemented algorithms, in terms of features and models. Generalizability has been defined as the algorithm's ability to generalize across tasks, sessions, or subjects with an accuracy higher than 80% (Zhou et al., 2021). In this work, we focused on cross-task generalizability. For all the targeted human factors, the water-based passive-BCI system has been calibrated using laboratory data. The design of appropriate training tasks for a cross-task approach is the main challenge since the tasks should be simple, short, and without confounding effects (Gerjets et al., 2014).

The obtained results showed that the water-based passive-BCI system calibrated cross-task allowed the discrimination between two levels of the targeted human factors. In particular, this approach applied to the workload monitoring reached an AUC higher than 0.8 at 40 s of temporal resolution providing the same performance as the intra-subject approach. Similar results have been obtained for the monitoring of vigilance. The intra-subject approach provided performances 10% higher than the cross-task approach, even if they are not significantly different. The highest values in terms of AUC have been reached during the stress monitoring: in both cases, the systems reached values higher than 0.95 after 20 s of temporal resolution. As said, these results are affected by the choice of the task for calibration, therefore the results indicated that the choice made for stress is as far better than those made for workload and vigilance.

The approach used here allowed a continuous estimate of the targeted human factors in terms of score (i.e., the probability of belonging to the high class), which can be considered the output index of the passive-BCI system. We have analyzed the similarity between the indices obtained through the intra-subject and the cross-subject approach. In particular, the stress indices were highly correlated to the population (R = 0.89), whereas they showed also higher distances (RMSE = 0.27) between the curves compared to the indices obtained for vigilance and workload. In contrast, the correlation between the workload and vigilance indices obtained with the intra-subject and cross-subject approach is medium (0.6 and 0.48, respectively). The temporal delay between the training and testing data may have disadvantaged the cross-task results. Future testing should involve cross-session recordings to better investigate this bias. Overall, the obtained results confirmed the appropriateness of the cross-task approach used for stress assessment, and that improvement should be achieved for workload and vigilance. In future work, different calibration tasks will be designed and tested on realistic driving.

Contextualizing these results in the literature, acceptable values of performance have been found only for cross-task calibrated and then tested on similar tasks. For example, Ke et al. (2015) reached an accuracy higher than 0.9 in mental workload classification using spatial and verbal n-back. In contrast, the study of Baldwin and Penaranda (2012) showed low accuracy in the cross-task classification using three different working memory tasks. The authors hypothesized that this result was due to the different neural structures underpinning each task. Therefore, when the cross-task procedure encompasses a predefined set of features, on the one hand, this can avoid the misclassification due to diverse concurrent mental states. On the other hand, a predefined set of features limits the generalizability of the cross-task approach. Therefore, in future attempts, the two approaches (predefined set of feature vs. features selection) should be compared.

#### Limitations and Future Work

One limitation of this study is related to the choice of performing simultaneous recording with the two kinds of electrodes. On the one hand, the simultaneous registration allows for avoiding temporal bias. On the other hand, there is a spatial bias that can affect the obtained correlation because they record different bioelectrical activities. The distance between the electrodes was about 1.5 cm to avoid electrical bridges due to the simultaneous presence of gel and water. A second limitation is related to the specificity of neurophysiological indices in realistic contexts. For example, visual-related activity during the task could hide a variation of parietal alpha due to workload. Also, systematic artifacts could be erroneously selected as features by the system leading to incorrect classification. Moreover, the current paper showed the system can discriminate between two levels of workload, stress, or vigilance. However, it is essential to move from a binary classification to multi-class classification. For example, the well-known inverted-U function of the Yerkes–Dodson law (Yerkes and Dodson, 1908) associated arousal with performance. According to this, it would be ideal to distinguish at least a suboptimal, an optimal, and an overload condition.

In the future, guidelines should be provided for the description of the tasks to be performed for effective cross-task calibration. The task in question must be simple to not require too much time for training, although it must be long enough to guarantee enough data for training the model. Finally, these water-based electrodes should be tested against already existing water electrodes (Volosyak et al., 2010; Schwarz et al., 2020).

## Conclusion

The main aim of this work was to test a new water-based passive BCI system for workload, vigilance, and stress monitoring for out-of-the-lab applications. The results showed that water electrodes guarantee higher ease of use, without lowering the performance compared to the gold standard. In addition, a proper structure for housing the EEG equipment would make the system wearable, so easy to fit and comfortable, and consequently acceptable for many different real-life applications. Moreover, the generalizability of the system, analyzed through a cross-task approach, showed acceptable performance according to different values of temporal resolution for each investigated human factor.

## Data Availability Statement

The datasets presented in this article are not readily available because of privacy restrictions. Requests to access the datasets should be directed to PA, pietro.arico@uniroma1.it.

## Ethics Statement

The studies involving human participants were reviewed and approved by Institutional Review Board of Sapienza University of Rome. The patients/participants provided their written informed consent to participate in this study.

## Author Contributions

Conceptualization and writing—original draft preparation: NS and PA. Methodology: NS, AD, and PA. Formal analysis: NS, DG, GD, AG, and PA. Software: AD. Investigation: NS, PA, GD, and GB. Resources and funding acquisition: FB. Data curation: GB and AV. Writing—review and editing: NS, PA, VR, AV, GD, DG, GB, and AG. Supervision: FB and PA. Project administration: FB, PA, GB, and GD. All authors contributed to the article and approved the submitted version.

## Funding

This work was co-financed by the European Commission by Horizon2020 projects MINDTOOTH: wearable device to decode human mind by neurometrics for a new concept of smart interaction with the surrounding environment (GA No. 950998). H2020-SESAR-2019-2 projects: Transparent artificial intelligence and automation to air traffic management systems, ARTIMATION, (GA No. 894238), WORKINGAGE: smart working environments for all Ages (GA No. 826232), FITDRIVE: monitoring devices for overall FITness of Drivers (GA No. 953432), SAFEMODE: Strengthening synergies between Aviation and maritime in the area of human Factors toward achieving more Efficient and resilient MODE of transportation (GA No. 814961), and BRAIN-SAFEDRIVE: a technology to detect Mental States during Drive for improving the Safety of the road (Italy-Sweden collaboration) with a grant of Ministero dell'Istruzione dell'Università e della Ricerca della Repubblica Italiana. The individual grants BRAIN2GETHER (BE-FOR-ERC) and NEUROSIM (Avvio alla ricerca 2020), recognized by Sapienza University of Rome to GD are also acknowledged.

## Conflict of Interest

NS, GD, DG, AG, AD, GB, AV, VR, FB, and PA were employed by BrainSigns Srl.

## Publisher's Note

All claims expressed in this article are solely those of the authors and do not necessarily represent those of their affiliated organizations, or those of the publisher, the editors and the reviewers. Any product that may be evaluated in this article, or claim that may be made by its manufacturer, is not guaranteed or endorsed by the publisher.

## References

[B1] Abdi-Sargezeh B. Foodeh R. Shalchyan V. Daliri M. R. (2021). EEG artifact rejection by extracting spatial and spatio-spectral common components. J. Neurosci. Methods 358, 109182. 10.1016/j.jneumeth.2021.109182 33836173

[B2] Alimardani M. Hiraki K. (2020). Passive brain-computer interfaces for enhanced human-robot. Interaction 7, 1–12. 10.3389/frobt.2020.00125 33501291 PMC7805996

[B3] Amaral C. P. Simões M. A. Mouga S. Andrade J. Castelo-Branco M. (2017). A novel brain computer interface for classification of social joint attention in autism and comparison of 3 experimental setups: a feasibility study. J. Neurosci. Methods 290, 105–115. 10.1016/j.jneumeth.2017.07.029 28760486

[B4] Aricò P. Borghini G. Di Flumeri G. Bonelli S. Golfetti A. Graziani I.. (2017b). Human factors and neurophysiological metrics in air traffic control: a critical review. IEEE Rev. Biomed. Eng. 10, 250–263. 10.1109/RBME.2017.2694142 28422665

[B5] Aricò P. Borghini G. Di Flumeri G. Colosimo A. Pozzi S. Babiloni F. (2016). A passive brain–computer interface application for the mental workload assessment on professional air traffic controllers during realistic air traffic control tasks. Prog. Brain Res. 228, 295–328. 10.1016/bs.pbr.2016.04.021 27590973

[B6] Aricò P. Borghini G. Di Flumeri G. Sciaraffa N. Babiloni F. (2018). Passive BCI beyond the lab: current trends and future directions. Physiol. Meas. 39, 08TR02. 10.1088/1361-6579/aad57e 30039806

[B7] Aricò P. Borghini G. Di Flumeri G. Sciaraffa N. Colosimo A. Babiloni F. (2017a). Passive BCI in operational environments: insights, recent advances, and future trends. IEEE Trans. Biomed. Eng. 64, 1431–1436. 10.1109/TBME.2017.2694856 28436837

[B8] Attallah O. (2020). An effective mental stress state detection and evaluation system using minimum number of frontal brain electrodes. Diagnostics 10, 292. 10.3390/diagnostics10050292 32397517 PMC7278014

[B9] Baldwin C. L. Penaranda B. N. (2012). Adaptive training using an artificial neural network and EEG metrics for within-and cross-task workload classification. NeuroImage 59, 48–56. 10.1016/j.neuroimage.2011.07.047 21835243

[B10] Bamber D. (1975). The area above the ordinal dominance graph and the area below the receiver operating characteristic graph. J. Math. Psychol. 12, 387–415.

[B11] Blankertz B. Acqualagna L. Dähne S. Haufe S. Schultze-Kraft M. Sturm I.. (2016). The Berlin brain-computer interface: progress beyond communication and control. Front. Neurosci. 10, 530. 10.3389/fnins.2016.00530 27917107 PMC5116473

[B12] Borghini G. Aricò P. Astolfi L. Toppi J. Cincotti F. Mattia D.. (2013). “Frontal EEG theta changes assess the training improvements of novices in flight simulation tasks,” in Proceedings of the 2013 35th Annual International Conference of the IEEE Engineering in Medicine and Biology Society (EMBC), 6619–6622. 10.1109/EMBC.2013.6611073 24111260

[B13] Borghini G. Astolfi L. Vecchiato G. Mattia D. Babiloni F. (2014). Measuring neurophysiological signals in aircraft pilots and car drivers for the assessment of mental workload, fatigue and drowsiness. Neurosci. Biobehav. Rev. 44, 58–75. 10.1016/j.neubiorev.2012.10.003 23116991

[B14] Borghini G. Di Flumeri G. Aricò P. Sciaraffa N. Bonelli S. Ragosta M.. (2020). A multimodal and signals fusion approach for assessing the impact of stressful events on air traffic controllers. Sci. Rep. 10, 1–18. 10.1038/s41598-020-65610-z 32451424 PMC7248090

[B15] Breiman L. (2001). Random forests. Mach. Learn. 45, 5–32. 10.1023/A:1010933404324

[B16] Chakladar D. Das Dey S. Roy P. P. Dogra D. P. (2020). EEG-based mental workload estimation using deep BLSTM-LSTM network and evolutionary algorithm. Biomed. Signal Process. Control 60, 101989. 10.1016/j.bspc.2020.101989

[B17] Chi Y. M. Wang Y. Te Wang Y. Maier C. Jung T. P. Cauwenberghs G. (2012). Dry and noncontact EEG sensors for mobile brain-computer interfaces. IEEE Trans. Neural Syst. Rehabil. Eng. 20, 228–235. 10.1109/TNSRE.2011.2174652 22180514

[B18] Choi H. S. Min S. Kim S. Bae H. Yoon J. E. Hwang I.. (2019). Learning-based instantaneous drowsiness detection using wired and wireless electroencephalography. IEEE Access 7, 146390–146402. 10.1109/ACCESS.2019.2946053

[B19] Comstock Jr. Raymond J. Arnegard R. J. (1992). The Multi-Attribute Task Battery for Human Operator Workload and Strategic Behavior Research. No. NAS 1.15: 104174.

[B20] Delorme A. Makeig S. (2004). EEGLAB: an open source toolbox for analysis of single-trial EEG dynamics including independent component analysis. J. Neurosci. Methods 134, 9–21. 10.1016/j.jneumeth.2003.10.009 15102499

[B21] Di Flumeri G. Aricò P. Borghini G. Colosimo A. Babiloni F. (2016). “A new regression-based method for the eye blinks artifacts correction in the EEG signal, without using any EOG channel,” in Conference Proceedings: Annual International Conference of the IEEE Engineering in Medicine and Biology Society. IEEE Engineering in Medicine and Biology Society Conference. 10.1109/EMBC.2016.7591406 28268985

[B22] Di Flumeri G. Aricò P. Borghini G. Sciaraffa N. Di Florio A. Babiloni F. (2019a). The dry revolution: evaluation of three different EEG dry electrode types in terms of signal spectral features, mental states classification and usability. Sensors 19, 1365. 10.3390/s19061365 30893791 PMC6470960

[B23] Di Flumeri G. Borghini G. Aricò P. Sciaraffa N. Lanzi P. Pozzi S.. (2018). EEG-based mental workload neurometric to evaluate the impact of different traffic and road conditions in real driving settings. Front. Hum. Neurosci. 12, 509. 10.3389/fnhum.2018.00509 30618686 PMC6305466

[B24] Di Flumeri G. De Crescenzio F. Berberian B. Ohneiser O. Kramer J. Aricò P.. (2019b). Brain–computer interface-based adaptive automation to prevent out-of-the-loop phenomenon in air traffic controllers dealing with highly automated systems. Front. Hum. Neurosci. 13, 296. 10.3389/fnhum.2019.00296 31555113 PMC6743225

[B25] Di Nardo M. D. Forino D. Murino T. (2020). The evolution of man–machine interaction: the role of human in Industry 4.0 paradigm. Prod. Manuf. Res. 8, 20–34. 10.1080/21693277.2020.1737592

[B26] Douibi K. Le Bars S. Lemontey A. Nag L. Balp R. Breda G. (2021). Toward EEG-based BCI applications for industry 4.0: challenges and possible applications. Front. Hum. Neurosci. 15, 705064. 10.3389/fnhum.2021.705064 34483868 PMC8414547

[B27] Edelberg R. (1972). Electrical activity of the skin: its measurement and uses in psychophysiology. Handbook Psychophysiol. 1972:367–418.18544832

[B28] Erp J. B. F. Van Lotte F. Tangermann M. (2012). Brain-computer interfaces: Beyond medical applications. Computer. 45, 26–34. 10.1109/MC.2012.107

[B29] Fan J. Wade J. W. Bian D. Key A. P. Warren Z. E. Mion L. C.. (2015). “A step towards EEG-based brain computer interface for autism intervention,” in Proceedings of the 2015 37th Annual International Conference of the IEEE Engineering in Medicine and Biology Society (EMBC). IEEE, 3767–3770. 26737113 10.1109/EMBC.2015.7319213 PMC5600898

[B30] Ferree T. C. Luu P. Russell G. S. Tucker D. M. (2001). Scalp electrode impedance, infection risk, and EEG data quality. Clin. Neurophysiol. 112, 536–544. 10.1016/S1388-2457(00)00533-2 11222977

[B31] Gerjets P. Walter C. Rosenstiel W. Bogdan M. Zander T. O. (2014). Cognitive state monitoring and the design of adaptive instruction in digital environments: lessons learned from cognitive workload assessment using a passive brain-computer interface approach. Front. Neurosci. 8, 385. 10.3389/fnins.2014.00385 25538544 PMC4260500

[B32] Gevins A. Smith M. E. McEvoy L. Yu D. (1997). High-resolution EEG mapping of cortical activation related to working memory: effects of task difficulty, type of processing, and practice. Cerebral Cortex 7, 374–385. 9177767 10.1093/cercor/7.4.374

[B33] Halim Z. Rehan M. (2020). On identification of driving-induced stress using electroencephalogram signals: A framework based on wearable safety-critical scheme and machine learning. Information Fusion 53, 66–79. 10.1016/j.inffus.2019.06.006

[B34] He H. Bai Y. Garcia E. A. Li S. (2008). ADASYN: adaptive synthetic sampling approach for imbalanced learning. Proc. Int. Joint Conf. Neural Netw. 3, 1322–1328. 10.1109/IJCNN.2008.4633969

[B35] Hekmatmanesh A. Zhidchenko V. Kauranen K. Siitonen K. Handroos H. Soutukorva S.. (2021). Biosignals in human factors research for heavy equipment operators: a review of available methods and their feasibility in laboratory and ambulatory studies. IEEE Access 9, 97466–97482. 10.1109/ACCESS.2021.3092516

[B36] Hernández L. G. Mozos O. M. Ferrández J. M. Antelis J. M. (2018). EEG-based detection of braking intention under different car driving conditions. Front. Neuroinform. 12, 1–14. 10.3389/fninf.2018.00029 29910722 PMC5992396

[B37] Hobfoll S. E. Shirom A. (1993). Stress and burnout in the workplace: conservation of resources. Handbook Organ. Behav. 1, 41–61.31500365

[B38] Hubbard J. Kikumoto A. Mayr U. (2019). EEG decoding reveals the strength and temporal dynamics of goal-relevant representations. Sci. Rep. 9, 1–11. 10.1038/s41598-019-45333-6 31227796 PMC6588723

[B39] Jamil N. Belkacem A. N. Ouhbi S. Lakas A. (2021). Noninvasive electroencephalography equipment for assistive, adaptive, and rehabilitative brain–computer interfaces: a systematic literature review. Sensors 21, 4754. 10.3390/s21144754 34300492 PMC8309653

[B40] Kamrud A. Borghetti B. Kabban C. S. Miller M. (2021). Generalized deep learning EEG models for cross-participant and cross-task detection of the vigilance decrement in sustained attention tasks. Sensors. 21, 5617. 10.3390/s21165617 PMC8402570 34451059

[B41] Kappenman E. S. Luck S. J. (2010). The effects of electrode impedance on data quality and statistical significance in ERP recordings. Psychophysiology 47, 888–904. 10.1111/j.1469-8986.2010.01009.x 20374541 PMC2902592

[B42] Katmah R. Al-Shargie F. Tariq U. Babiloni F. Al-Mughairbi F. Al-Nashash H. (2021). A review on mental stress assessment methods using eeg signals. Sensors 21, 5043. 10.3390/s21155043 34372280 PMC8347831

[B43] Ke J. Zhang M. Luo X. Chen J. (2021). Monitoring distraction of construction workers caused by noise using a wearable electroencephalography (EEG) device. Autom. Const. 125, 103598. 10.1016/j.autcon.2021.103598

[B44] Ke Y. Qi H. Zhang L. Chen S. Jiao X. Zhou P.. (2015). Towards an effective cross-task mental workload recognition model using electroencephalography based on feature selection and support vector machine regression. Int. J. Psychophysiol. 98, 157–166. 10.1016/j.ijpsycho.2015.10.004 26493860

[B45] Klimesch W. (1999). EEG alpha and theta oscillations reflect cognitive and memory performance: a review and analysis. Brain Res. Brain Res. Rev. 29, 169–195. 10209231 10.1016/s0165-0173(98)00056-3

[B46] Li G. Chung W.-Y. (2022). Electroencephalogram-based approaches for driver drowsiness detection and management: a review. Sensors 22:1100. 10.3390/s22031100 35161844 PMC8840041

[B47] Lim W. L. Sourina O. Wang L. P. (2018). STEW: simultaneous task EEG workload data set. IEEE Trans. Neural Syst. Rehabil. Eng. 26, 2106–2114. 10.1109/TNSRE.2018.2872924 30281467

[B48] Lin C. T. Chuang C. H. Huang C. S. Tsai S. F. Lu S. W. Chen Y. H.. (2014). Wireless and wearable EEG system for evaluating driver vigilance. IEEE Trans. Biomed. Circuits Syst. 8, 165–176. 10.1109/TBCAS.2014.2316224 24860041

[B49] Lopez-Gordo M. A. Sanchez Morillo D. Pelayo Valle F. (2014). Dry EEG electrodes. Sensors 14, 12847–12870. 10.3390/s140712847 25046013 PMC4168519

[B50] Makeig S. Bell A. Jung T.-P. Sejnowski T. J. (1995). Independent component analysis of electroencephalographic data. Adv. Neural Inform. Process. Syst. 8, 145–151.14622887

[B51] Mehreen A. Anwar S. M. Haseeb M. Majid M. Ullah M. O. (2019). A hybrid scheme for drowsiness detection using wearable sensors. IEEE Sens. J. 19, 5119–5126. 10.1109/JSEN.2019.2904222

[B52] Molina E Correa Á. Sanabria D. Jung T. P. (2013). “Tonic EEG dynamics during psychomotor vigilance task,” in Proceedings of the 2013 6th International IEEE/EMBS Conference on Neural Engineering (NER), 1382–1385. 10.1109/NER.2013.6696200

[B53] Molina E. Sanabria D. Jung T.-P. Correa A. (2019). Electroencephalographic and peripheral temperature dynamics during a prolonged psychomotor vigilance task. Accid. Anal. Prevent. 126, 198–208. 10.1016/j.aap.2017.10.014 29061281

[B54] Müller-Putz G. Wriessnegger S. C. (2021). “Electroencephalography and brain–computer interfaces,” in Neuroprosthetics and Brain-Computer Interfaces in Spinal Cord Injury (New York, NY: Springer), 71–103. 10.1007/978-3-030-68545-4_3

[B55] Neigel A. R. Claypoole V. L. Smith S. L. Waldfogle G. E. Fraulini N. W. Hancock G. M.. (2020). Engaging the human operator: a review of the theoretical support for the vigilance decrement and a discussion of practical applications. Theor. Issues Ergon. Sci. 21, 239–258. 10.1080/1463922X.2019.1682712

[B56] Noreika V. Georgieva S. Wass S. Leong V. (2020). 14 challenges and their solutions for conducting social neuroscience and longitudinal EEG research with infants. Infant Behav. Develop. 58, 101393. 10.1016/j.infbeh.2019.101393 31830682

[B57] Parasuraman R. Warm J. S. See J. E. (1998). “Brain systems of vigilance,” in The Attentive Brain (Cambridge, MA: The MIT Press), 221–256.

[B58] Roman-Gonzalez A. (2012). “Eeg signal processing for BCI applications,” in Human–Computer Systems Interaction: Backgrounds and Applications 2 (New York, NY: Springer), 571–591. 10.1007/978-3-642-23187-2_36

[B59] Roy R. N. Charbonnier S. Campagne A. Bonnet S. (2016). Efficient mental workload estimation using task-independent EEG features. J. Neural Eng. 13, 26019. 10.1088/1741-2560/13/2/026019 26877162

[B60] Ruffini G. Dunne S. Fuentemilla L. Grau C. Farres E. Marco-Pallarés J.. (2008). First human trials of a dry electrophysiology sensor using a carbon nanotube array interface. Sens. Actuat. A Phys. 144, 275–279. 10.1016/j.sna.2008.03.007

[B61] Saeed S. M. U. Anwar S. M. Majid M. Bhatti A. M. (2015). “Psychological stress measurement using low cost single channel EEG headset,” in Proceedings of the 2015 IEEE International Symposium on Signal Processing and Information Technology (ISSPIT). IEEE, 581–585. 10.1109/ISSPIT.2015.7394404

[B62] Schaffer C. (1993). Selecting a classification method by cross-validation. Mach. Learn. 13, 135–143.

[B63] Schultze-Kraft M. Dähne S. Gugler M. Curio G. Blankertz B. (2016). Unsupervised classification of operator workload from brain signals. J. Neural Eng. 13, 36008. 10.1088/1741-2560/13/3/036008 27078889

[B64] Schwarz A. Escolano C. Montesano L. Müller-Putz G. R. (2020). Analyzing and decoding natural reach-and-grasp actions using gel, water and dry EEG systems. Front. Neurosci. 14, 1–17. 10.3389/fnins.2020.00849 32903775 PMC7438923

[B65] Sciaraffa N. Borghini G. Di Flumeri G. Cincotti F. Babiloni F. Aricò P. (2021). Joint analysis of eye blinks and brain activity to investigate attentional demand during a visual search task. Brain Sci. 11, 562. 10.3390/brainsci11050562 33925209 PMC8146019

[B66] Sciaraffa N. Flumeri G. Di Germano D. Giorgi A. Florio A. Di Borghini G.. (2022). Validation of a light EEG-based measure for real-time stress monitoring during realistic driving. Brain Sci. 12:304. 10.3390/brainsci12030304 35326261 PMC8946850

[B67] Sebastiani M. Di Flumeri G. Aricò P. Sciaraffa N. Babiloni F. Borghini G. (2020). Neurophysiological vigilance characterisation and assessment: laboratory and realistic validations involving professional air traffic controllers. Brain Sci. 12, 304. 10.3390/brainsci10010048 PMC7016567 31952181

[B68] Seo S.-H. Lee J.-T. (2010). Stress and EEG. Convergence and Hybrid Information Technologies. Vienna: IntechOpen. 10.5772/9651

[B69] Skoluda N. Strahler J. Schlotz W. Niederberger L. Marques S. Fischer S.. (2015). Intra-individual psychological and physiological responses to acute laboratory stressors of different intensity. Psychoneuroendocrinology 51, 227–236. 10.1016/j.psyneuen.2014.10.002 25462896

[B70] Somers B. Francart T. Bertrand A. (2018). A generic EEG artifact removal algorithm based on the multi-channel Wiener filter. J. Neural Eng. 15, 36007. 10.1088/1741-2552/aaac92 29393057

[B71] Stanton N. A. Young M. S. (2000). A proposed psychological model of driving automation. Theor. Issues Ergon. Sci. 1, 315–331. 10.1080/14639220052399131

[B72] Tautan A.-M. Mihajlovic V. Chen Y.-H. Grundlehner B. Penders J. Serdijn W. A. (2014). “Signal quality in dry electrode EEG and the relation to skin-electrode contact impedance magnitude,” in Biodevices, 12–22.

[B73] Tǎutan A. M. Mihajlović V. Chen Y. H. Grundlehner B. Penders J. Serdijn W. (2014). “Signal quality in dry electrode EEG and the relation to skin-electrode contact impedance magnitude,” in BIODEVICES 2014 7th International Conference on Biomedical Electronics and Devices, Proceedings; Part of 7th International Joint Conference on Biomedical Engineering Systems and Technologies, BIOSTEC 2014, 12–22.

[B74] Topor M. Opitz B. Dean P. J. A. (2021). In search for the most optimal EEG method: a practical evaluation of a water-based electrode EEG system. Brain Neurosci. Adv. 5, 239821282110536. 10.1177/23982128211053698 34722932 PMC8554570

[B75] Toyama S. Takano K. Kansaku K. (2012). A non-adhesive solid-gel electrode for a non-invasive brain–machine interface. Front. Neurol. 3, 114. 10.3389/fneur.2012.00114 22826701 PMC3399135

[B76] Volosyak I. Valbuena D. Malechka T. Peuscher J. Gräser A. (2010). Brain-computer interface using water-based electrodes. J. Neural Eng. 7, 066007. 10.1088/1741-2560/7/6/066007 21048286

[B77] Vourvopoulos A. Niforatos E. Hlinka M. Skola F. Liarokapis F. (2017). “Investigating the effect of user profile during training for BCI-based games,” in Proceedings of the 2017 9th International Conference on Virtual Worlds and Games for Serious Applications (VS-Games). IEEE, 117–124. 10.1109/VS-GAMES.2017.8056579

[B78] Webster J. G. (2009). Medical Instrumentation: Application and Design. Hoboken, NJ: Wiley.

[B79] Wei C. S. Wang Y. Te Lin C. T. Jung T. P. (2018). Toward drowsiness detection using non-hair-bearing EEG-based brain-computer interfaces. IEEE Trans. Neural Syst. Rehabil. Eng. 26, 400–406. 10.1109/TNSRE.2018.2790359 29432111

[B80] Wetherell M. A. Sidgreaves M. C. (2005). Secretory immunoglobulin-A reactivity following increases in workload intensity using the Defined Intensity Stressor Simulation (DISS). Stress Health J. Int. Soc. Investig. Stress 21, 99–106. 10.1002/smi.1038

[B81] Wickens C. D. (2008). Multiple resources and mental workload. Hum. Fact. 50, 449–455. 10.1518/001872008X288394 18689052

[B82] Wilkinson R. T. Houghton D. (1982). Field test of arousal: a portable reaction timer with data storage. Hum. Fact. 24, 487–493. 7129455 10.1177/001872088202400409

[B83] Yerkes R. M. Dodson J. D. (1908). The relation of strength of stimulus to rapidity of habit-formation. Punish. Issues Exp. 1908, 27–41.

[B84] Young M. S. Brookhuis K. A. Wickens C. D. Hancock P. A. (2015). State of science: mental workload in ergonomics. Ergonomics 58, 1–17. 10.1080/00140139.2014.956151 25442818

[B85] Zander T. O. Kothe C. (2011). Towards passive brain–computer interfaces: applying brain–computer interface technology to human–machine systems in general. J. Neural Eng. 8, 25005. 10.1088/1741-2560/8/2/025005 21436512

[B86] Zander T. O. Lehne M. Ihme K. Jatzev S. Correia J. Kothe C.. (2011). A dry EEG-system for scientific research and brain-computer interfaces. Front. Neurosci. 5, 1–10. 10.3389/fnins.2011.00053 21647345 PMC3103872

[B87] Zander T. O. Phypa T. Jatzev S. (2009). Detecting affective covert user states with passive. Brain–Computer Interf. 2, 54. 10.1109/ACII.2009.5349456

[B88] Zander T. O. Shetty K. Lorenz R. Leff D. R. Krol L. R. Darzi A.. (2017). Automated task load detection with electroencephalography: towards passive brain–computer interfacing in robotic surgery. J. Med. Robot. Res. 2, 1750003. 10.1142/S2424905X17500039

[B89] Zhou Y. Huang S. Xu Z. Wang P. Wu X. Zhang D. (2021). Cognitive workload recognition using EEG signals and machine learning: a review. IEEE Trans. Cogn. Develop. Syst. 8920, 1. 10.1109/TCDS.2021.3090217

